# Cross-modal conditional associations between psychometric and polysomnographic indicators in sleep center patients: a Gaussian graphical model

**DOI:** 10.3389/fpsyt.2026.1907602

**Published:** 2026-09-03

**Authors:** Yuting Wang, Na Ni, Hui Chen, Jiahao Zhao, Yanqing Xie, Bixia Zhao, Fengying Zhang

**Affiliations:** 1 Sleep Medicine Center, Department of Neurology, The Affiliated Nanhua Hospital, Hengyang Medical School, University of South China, Hengyang, China; 2 School of Nursing, The Affiliated Nanhua Hospital, Hengyang Medical School, University of South China, Hengyang, China

**Keywords:** cross-modal conditional association, Gaussian graphical model, latent profile analysis, multimodal network analysis, polysomnography, psychometric assessment, subjective-objective sleep discrepancy

## Abstract

**Background:**

Subjective-objective sleep discrepancy is widely recognized in sleep medicine, but the multivariable relationships between psychometric assessments and polysomnographic (PSG) parameters remain insufficiently understood. Previous network studies in sleep populations have relied primarily on subjective scales or questionnaire items. This study estimated a Gaussian graphical model (GGM) integrating psychometric and PSG indicators to examine their cross-modal conditional association structure.

**Methods:**

We analyzed data from 318 sleep center patients who underwent overnight PSG and routine psychometric assessment. Eighteen indicators were included: two Hamilton Anxiety Rating Scale factors, seven 24-item Hamilton Depression Rating Scale factors, four Dysfunctional Beliefs and Attitudes about Sleep Scale factors, and five PSG parameters comprising sleep efficiency, apnea-hypopnea index, arousal index, N3 percentage, and rapid eye movement sleep percentage. Redundancy screening removed one psychometric factor before network estimation, leaving 17 network nodes. The analyses included latent profile analysis with comparison of all available covariance parameterizations, an EBICglasso-estimated GGM, network comparison tests, and moderated network analysis.

**Results:**

Latent profile analysis identified five patient profiles (entropy = 0.897), including two small profiles with opposite patterns: one combined markedly elevated anxiety and depression indicators with near-average PSG values, and the other combined clearly abnormal respiratory and arousal indicators with lower psychometric scores. The estimated GGM contained 43 nonzero edges out of 136 possible, including three small cross-modal edges, all of which were negative (mean weight = −0.027). Psychic anxiety had the highest expected influence (1.134), whereas four of the five PSG indicators occupied the four lowest ranks. The case-dropping bootstrap yielded a correlation stability coefficient of.673. Psychic anxiety (−0.038) and log(AHI + 1) (−0.038) had the largest-magnitude bridge expected influence values within the psychometric and PSG modalities, followed by medication beliefs (−0.034). Network comparison tests did not detect statistically significant differences in network structure (M = 0.187, p = .808) or global strength (S = 0.304, p = .819) between the two largest profiles. No nonzero moderation parameter involving apnea-hypopnea index was retained by the regularized moderated network model.

**Conclusions:**

In this cross-sectional clinical sample, the estimated network was characterized by predominantly stronger within-modality associations and limited cross-modal conditional associations between psychometric and PSG indicators. These findings suggest that psychometric and PSG assessments may capture partly non-overlapping and complementary aspects of patients’ clinical presentations. However, the observed network structure does not establish mechanistic decoupling, causal direction, or independence between psychological and physiological processes. Longitudinal studies with closer temporal alignment and repeated PSG measurements are needed to determine the sources and clinical significance of these cross-modal associations.

## 1 Introduction

Sleep center populations are diagnostically heterogeneous. Obstructive sleep apnea alone affects an estimated 936 million adults worldwide (1), chronic insomnia has a global prevalence of approximately 16% (2), and the two conditions frequently co-occur, with 30% to 50% of OSA patients reporting clinically significant insomnia symptoms (3). Psychological distress is pervasive in these patients. A meta-analysis of 73 studies estimated the pooled prevalence of depressive and anxiety symptoms in OSA patients at 35% and 32%, respectively (4), and insomnia itself is an independent predictor of subsequent depression (5). Beyond affective symptoms, dysfunctional beliefs about sleep contribute to the maintenance of sleep-related distress through cognitive processes such as selective attention and worry (6). How these multiple dimensions of subjective psychological experience relate to objective polysomnographic findings remains a persistent clinical question.

Subjective-objective sleep discrepancy is well documented in sleep medicine. Insomnia patients consistently overestimate sleep onset latency and underestimate total sleep time relative to polysomnographic recordings (7). A meta-analysis of PSG studies confirmed that insomnia patients do exhibit measurable sleep abnormalities, but effect sizes are small relative to the severity of subjective complaints (8). In extreme cases, patients report severe insomnia despite normal PSG findings, a phenomenon termed paradoxical insomnia (9). This discrepancy is not limited to the insomnia direction. Among OSA patients, greater apnea severity is associated with lower levels of anxiety and depression (10), indicating that objective severity and subjective distress can move in opposite directions. However, existing evidence relies primarily on bivariate correlations or group comparisons, which provide limited information about how associations change after accounting for other measured clinical indicators. In the present study, subjective-objective discrepancy refers to potential divergence between psychometric and PSG findings and is not treated as a directly measured construct or as evidence of mechanistic decoupling between psychological and physiological processes.

Network analysis offers a multivariable framework for examining these relationships. Gaussian graphical models (GGMs) estimate partial correlations among all variables, representing undirected conditional associations after statistically controlling for every other node in the network. Unlike conventional total-score correlations, this approach estimates the association between each pair of variables conditional on all other included nodes. These associations do not establish temporal precedence, causal direction, mediation, or mechanistic relationships.

Network analysis has been increasingly applied in sleep medicine. Liu et al. (11) constructed a symptom network of anxiety, depression, and sleep quality in 1,571 insomnia patients using the HAMA, HAMD, and PSQI. Xing et al. (12) applied moderated network analysis to examine the role of fatigue in the insomnia-anxiety-depression interaction. Luo et al. (13) built an item-level network of anxiety, depression, and sleep disturbance symptoms in OSA patients. However, previous network analyses in the sleep domain have primarily included psychometric scale scores or questionnaire symptoms as nodes. To our knowledge, no previous sleep-network study has incorporated PSG parameters as nodes alongside psychometric indicators, leaving their cross-modal conditional association structure insufficiently characterized.

The gap extends beyond node selection. The relationship between dysfunctional sleep beliefs and PSG parameters itself yields conflicting evidence. Morin et al. (14) reported that DBAS-16 total scores were not significantly correlated with any PSG parameter. Yet Hertenstein et al. (15) found a weak negative association between DBAS total scores and PSG-determined total sleep time, suggesting that the relationship varies with analytic approach and sample composition. This inconsistency likely reflects the aggregation inherent in total scores. As Fried and Nesse (16) argued, sum scores compress heterogeneous subcomponents into a single value, obscuring component-level associations. The four DBAS factors do differ in clinical function: the medication beliefs subscale, for instance, is insensitive to cognitive behavioral therapy for insomnia (17). Modeling the four DBAS factors separately may reveal conditional associations that are obscured by the total score. The present study addresses both methodological gaps by jointly modeling four DBAS factors, two HAMA factors, seven HAMD factors, and five PSG parameters, from which the final network node set was derived after redundancy screening. To our knowledge, this is the first sleep-network study to incorporate PSG parameters alongside psychometric indicators.

Because these variables represent different measurement modalities, they were treated as heterogeneous clinical indicators rather than as interchangeable symptoms or measures of a single latent construct. Psychometric and PSG indicators were jointly modeled because they capture clinically complementary aspects of the same sleep-center presentation. The joint model addressed whether psychometric–PSG associations remained after accounting simultaneously for the other indicators from both modalities; estimating the modalities separately would exclude such cross-modal associations by design. Accordingly, the shared network space was defined as a joint patient-level statistical dependence space, rather than a common symptom ontology or latent construct, with nodes representing observed variables and edges representing conditional associations (18). Extended network analyses have likewise examined associations spanning heterogeneous mental and physical health domains (19), although such applications do not establish measurement equivalence. Joint estimation did not assume equivalence in construct, reliability, error structure, distribution, or temporal reference period across modalities. We therefore refer to the resulting model as a multimodal clinical indicator network.

Building on these two methodological gaps, we adopted a four-layer analytic framework. Latent profile analysis (LPA) identified patient subgroups based on all 18 indicators. A Gaussian graphical model estimated with EBICglasso mapped the full-sample network structure. Network comparison tests (NCT) examined whether network topology differed between the two largest profiles. Moderated network analysis (MNA) explored whether edge estimates varied as a function of AHI.

The present study used an exploratory, multimethod framework to characterize conditional associations among psychometric and polysomnographic indicators in a heterogeneous sleep-center sample. Specifically, we pursued four research aims: (1) describe the community structure and cross-modal conditional associations within the resulting indicator network; (2) examine the relative expected influence and bridge expected influence of the included indicators; (3) explore whether the estimated network structure differed between the two largest empirically derived latent profiles; and (4) explore whether AHI moderated pairwise conditional associations. These analyses were intended to generate hypotheses regarding cross-modal organization rather than to test confirmatory predictions.

## 2 Methods

### 2.1 Study design and setting

This was a single-center, retrospective, cross-sectional study conducted at the Sleep Medicine Center of the Affiliated Nanhua Hospital, University of South China. No prospective participant recruitment or random sampling was performed. The study used a non-probability, criterion-based retrospective sampling approach and included all available clinical records in the study dataset that met the predefined eligibility criteria during the specified study period. We integrated two types of clinical data from patients who underwent overnight polysomnography (PSG) at the center between January 2024 and April 2026: standardized psychometric scales collected during routine clinical assessment, and PSG monitoring records. The sample size was determined by the number of eligible clinical records available within the defined study period rather than by prospective statistical planning. No *a priori* sample-size calculation or simulation-based power analysis was conducted, and neither the study protocol nor the analysis plan was preregistered. This study was approved by the Medical Ethics Committee of the Affiliated Nanhua Hospital, University of South China (approval number: 2026-ky-013). The requirement for individual informed consent was waived due to the retrospective and de-identified nature of the data.

### 2.2 Participants

From the available clinical dataset, all records meeting the following predefined eligibility criteria were included: (1) aged 18 years or older, (2) completed the 16-item Dysfunctional Beliefs and Attitudes about Sleep Scale (DBAS-16), the Hamilton Anxiety Rating Scale (HAMA), and the 24-item Hamilton Depression Rating Scale (HAMD-24) during routine clinical assessment, (3) underwent overnight PSG within 7 days of the psychometric evaluation, and (4) had completed HAMA, HAMD-24, and DBAS-16 records with no missing items. Patients were excluded if total sleep time was below 120 minutes, as such recordings do not yield reliable sleep architecture data, or if key PSG parameters were missing. No threshold was imposed on the apnea-hypopnea index (AHI); the sample therefore included patients across the full range of sleep-disordered breathing severity, including those with a normal AHI (Section 3.1). Within the available 327-patient PSG analytic-source file, all patients had completed records for the three psychometric assessments. Four patients were excluded because total sleep time was below 120 minutes and five because at least one key PSG parameter was missing, leaving 318 patients in the final analytic sample.

### 2.3 Measures

#### 2.3.1 Psychometric scales

All three psychometric assessments were conducted face to face by trained healthcare professionals using a hospital-based electronic assessment system rather than paper-and-pencil forms. Patient responses to the DBAS-16 and clinician ratings for the HAMA and HAMD-24 were entered directly into the system during the assessment, after which the completed records were automatically scored and stored electronically for subsequent export. Incomplete assessment attempts could remain stored in the electronic system but were not finalized or scored. No item-level imputation was performed. An unfinished attempt did not itself lead to exclusion when the same patient had a completed record for that instrument within the prespecified 7-day window. The electronic system required a response or rating for every item before an assessment could be finalized and a score report generated. For the present study, only records designated as completed in the system were included in the data export.

The HAMA contains 14 items scored from 0 to 4, yielding a theoretical total-score range of 0–56 and two factor scores, psychic anxiety and somatic anxiety, each with a theoretical range of 0–28 (20). The HAMD-24 contains 24 items scored from 0 to 2 or from 0 to 4 and is organized into seven factors: anxiety/somatization, weight, cognitive impairment, diurnal variation, retardation, sleep disturbance, and hopelessness, with theoretical ranges of 0–18, 0–2, 0–22, 0–2, 0–14, 0–6, and 0–12, respectively; the theoretical total-score range is 0–76 (21). The DBAS-16 contains 16 items grouped into four factors: consequences, worry/helplessness, expectations, and medication (14). In the hospital-based electronic assessment system, each DBAS-16 item was scored from 1 to 5, yielding a theoretical total-score range of 16–80 and a theoretical range of 1–5 for each factor mean. Higher original DBAS-16 scores indicated more rational sleep beliefs, opposite to the direction of the HAMA and HAMD, in which higher scores indicated greater symptom severity. All DBAS-16 item scores were directionally reversed before analysis; the theoretical score ranges remained unchanged, but higher values then represented more dysfunctional sleep beliefs. All 13 psychometric indicators therefore share a uniform direction, with higher values indicating greater symptom severity or more dysfunctional beliefs.

#### 2.3.2 Polysomnographic parameters

Overnight PSG was performed using a standard montage and scored by trained polysomnographic technicians following the American Academy of Sleep Medicine scoring manual (22). Five parameters were extracted from PSG reports as PSG indicators: sleep efficiency (SE), apnea-hypopnea index (AHI), arousal index (ArI), percentage of N3 sleep (N3%), and percentage of REM sleep (REM%). The raw AHI distribution included two exact zero values (0.6%) and showed marked positive skewness (skewness = 1.74). A fixed log(AHI + 1) transformation was applied to retain zero values and reduce right skewness (transformed skewness = −0.28), after which the transformed values were Z-standardized for analysis. The additive constant was fixed at 1 in the original analysis and was not estimated from the present data. Together with the 13 psychometric factor scores, these five parameters formed a set of 18 indicators. The latent profile analysis used all 18 indicators, whereas the network node set was finalized at 17 indicators after the redundancy screening described in Section 2.4.3.

### 2.4 Statistical analysis

#### 2.4.1 Data preprocessing and reliability assessment

Psychometric data were matched to PSG records by patient name. When a patient had multiple PSG recordings, the one closest in date to the psychometric evaluation was selected. After reverse coding and log-transformation as described above, all 18 indicators were converted to Z-scores to place them on a common numerical scale. The latent profile analysis used all 18 standardized indicators, and the network analyses used the 17 indicators retained after redundancy screening. This standardization did not imply equivalence in construct, reliability, error structure, distribution, or temporal reference period across modalities.

Internal consistency was evaluated using item-level data from the same 318 participants included in the final analytic sample. Because the questionnaire items were ordinal, polychoric correlation matrices were used to estimate ordinal McDonald’s omega and 95% confidence intervals for the complete HAMA-14, HAMD-24, and DBAS-16 and for each factor containing three or more items that was used as a network indicator. For factors comprising two items, the Spearman–Brown coefficient was reported instead. Internal consistency was not estimated for the single-item HAMD-24 weight and diurnal-variation factors and was designated as not applicable. For the clinician-rated HAMA and HAMD-24, these coefficients were interpreted as measures of internal consistency among item scores rather than as estimates of inter-rater reliability.

For each indicator retained in the final primary network, we calculated the number of observations and missing values, mean, standard deviation, median, interquartile range, minimum, maximum, number and proportion of zero values, number of unique values, skewness, and excess kurtosis. Empirical distributions were examined using histograms or frequency plots according to the discreteness and zero concentration of each variable. Raw AHI and log(AHI + 1) were summarized and displayed separately.

All analyses were performed in R 4.5.1 (23); specific packages and versions are noted in the corresponding sections below.

#### 2.4.2 Latent profile analysis

Latent profile analysis was performed on the 18 Z-standardized indicators. Gaussian mixture models with k = 1 to 6 profiles were fitted using the mclust package version 6.1.2 (24). All 14 covariance parameterizations available in mclust were fitted, and model selection was restricted to the six parameterizations with diagonal covariance matrices (EII, VII, EEI, VEI, EVI, and VVI). This restriction follows from the local independence assumption that defines latent profile analysis, under which indicators are mutually independent conditional on profile membership; the ellipsoidal parameterizations estimate within-class covariances explicitly and therefore define classes partly by patterns of association among indicators rather than by their levels. The number of profiles and the covariance structure were determined in two steps. Solutions were first required to converge without singular covariance estimates or empty classes and to meet both an entropy of at least 0.80 and a smallest class proportion of at least 5%. Among the diagonal-family solutions meeting these requirements, the one with the lowest BIC was selected. These thresholds were not relaxed during model selection. Variables used to derive the latent profiles and their direct derivatives were summarized descriptively. Inferential comparisons were restricted to age, sex, and total sleep time, which were not included in the LPA, using one-way ANOVA with partial eta squared and *post hoc* pairwise comparisons, or chi-square tests with Cramér’s V, as appropriate. Residual distributions, variance homogeneity, and extreme values were examined before selecting between ordinary ANOVA with Tukey-adjusted comparisons and Welch ANOVA with Games-Howell comparisons.

#### 2.4.3 Gaussian graphical model and centrality analysis

Before network estimation, potential redundancy among all 78 pairs of the 13 psychometric indicators was assessed within the full 18-indicator dataset using the goldbricker function in the networktools package version 1.6.0, with p = 0.05, method = “hittner2003,” threshold = 0.25, and corMin = 0.50 (25). Pairs meeting this operational criterion were treated as candidate redundant pairs and reviewed against factor content and construct definitions before any decision to retain, combine, or remove a node.

The network was estimated using the bootnet package version 1.8 (26). The zero-order input correlation matrix was computed using the cor_auto procedure. All 17 indicators entered cor_auto as non-integer numeric Z-scores, and none was specified or detected as an ordered variable under the default ordinal-detection rule. Consequently, all 136 pairwise entries in the input correlation matrix were Pearson product-moment correlations; no polychoric or polyserial correlations were selected. The network was subsequently estimated from this correlation matrix using EBICglasso. The EBIC hyperparameter gamma was set to 0.50. Gamma governs the additional EBIC penalty applied to model complexity, with larger values generally favoring more conservative and sparser selected networks; it is a prespecified model-selection hyperparameter rather than a network parameter estimated from the data. The resulting network edges represent L1-regularized partial correlations rather than zero-order Pearson correlations. The sampling accuracy of individual edge-weight estimates was assessed using 1,000 nonparametric bootstrap samples. In each bootstrap iteration, participants were resampled with replacement, and the network was re-estimated using the same data-processing procedure, correlation-estimation method, estimator, and hyperparameter as in the primary analysis. Bootstrapped 95% confidence intervals were examined to characterize estimation uncertainty, with wider intervals indicating lower precision. These intervals were not interpreted as conventional significance tests against zero, and whether an interval included zero was not used to determine whether a regularized edge was present or absent. Because both positive and negative edges were expected in the network, we used Expected Influence (EI) rather than strength centrality to quantify node importance (27). To quantify cross-modal connectivity, we specified two *a priori* modality groups comprising 12 psychometric factor scores and five PSG parameters, and computed Bridge Expected Influence using the networktools package version 1.6.0 (25). The two groups were defined operationally by measurement modality for the calculation of Bridge Expected Influence and were not treated as empirically identified symptom communities. Because the groups differed in size, Bridge Expected Influence values were interpreted primarily within modality rather than as directly comparable measures of importance across modalities. We also applied the Walktrap and Spinglass algorithms for data-driven community detection and compared the resulting partitions with the prespecified two-modality grouping using normalized mutual information (NMI). Walktrap was run on the absolute edge weights, and Spinglass used the signed edge weights. Separately, the stability of Expected Influence estimates was assessed using a case-dropping bootstrap with 1,000 replications. Ten equally spaced case-dropping proportions were examined: 5.0%, 12.8%, 20.6%, 28.3%, 36.1%, 43.9%, 51.7%, 59.4%, 67.2%, and 75.0%, corresponding to retention of 95.0%, 87.2%, 79.4%, 71.7%, 63.9%, 56.1%, 48.3%, 40.6%, 32.8%, and 25.0% of cases. Owing to integer rounding in the sample of 318 participants, the realized retained sample sizes were 302, 277, 253, 228, 203, 178, 154, 129, 104, and 80. The CS coefficient was defined as the maximum proportion of cases that could be dropped while retaining, with 95% probability, a correlation of at least 0.70 between the subset and original Expected Influence estimates. Values below 0.25 were considered unsuitable for interpretation, whereas values above 0.50 were considered preferable (26). The network was visualized with a Fruchterman-Reingold layout using the qgraph package version 1.9.8 (28).

#### 2.4.4 Network comparison test

The network comparison test (NCT) was used to examine differences in network structure across latent profiles (29). The primary comparison was between the two largest profiles, C5 (n = 117) and C2 (n = 115). Subgroup networks were estimated with EBICglasso using gamma = 0, corresponding to BIC-based selection along the graphical-lasso regularization path; this setting did not remove graphical-lasso regularization. A total of 1,000 permutations were performed. Four aspects were tested: global strength invariance, network structure invariance, individual edge differences, and centrality differences. Benjamini-Hochberg correction was applied to the p values for individual edges, and expected influence was the centrality index tested. Because the five profiles differ along several dimensions and do not form a single ordering from lower to higher psychometric burden, profiles were not pooled for a supplementary comparison. All analyses were conducted using the NetworkComparisonTest package version 2.2.3. These comparisons evaluated network differences between the assigned groups and were not designed to assess the stability or validity of the five-profile solution.

#### 2.4.5 Moderated network analysis

To explore whether edge estimates among the remaining indicators varied as a function of sleep-disordered breathing severity, we performed a moderated network analysis using the mgm package version 1.2-15 (30). The Z-standardized log(AHI + 1) value served as the continuous moderator, with the remaining 16 nodes of the final node set as network variables. Model parameters were selected via 10-fold cross-validation with the AND rule. Conditional networks were estimated at three AHI levels: one standard deviation below the mean, the mean, and one standard deviation above the mean.

#### 2.4.6 Sensitivity analyses

The sensitivity of the network results to the estimation settings was evaluated in three ways. First, to assess sensitivity to node selection, we removed HD_F2 (weight) and HD_F4 (diurnal variation), which showed pronounced floor effects with 73.9% and 59.7% of patients scoring zero, and returned HD_F1, which had been excluded from the final node set for content overlap. The network was re-estimated with the resulting 16 nodes. Second, to assess sensitivity to the EBIC model-selection hyperparameter, the network was re-estimated with gamma = 0, corresponding to BIC-based selection along the graphical-lasso regularization path rather than removal of regularization. Third, to assess sensitivity to the marginal distributions and to the fixed AHI transformation, the network was re-estimated from a Spearman correlation matrix using the ranks of the untransformed AHI, with the same node set and gamma as in the primary analysis. Each network was compared with the primary network in terms of the number and density of non-zero edges, the correlation between the full edge weight vectors, the mean absolute difference, the Jaccard coefficient, sign agreement across shared non-zero edges, the number of cross-modal edges, and rank correlations for expected influence and for bridge expected influence within each modality.

To assess sensitivity to the interval between psychometric assessment and PSG, the network was re-estimated separately in the ≤3-day subsample (N = 312) and the ≤1-day subsample (N = 258). Each subsample was restandardized within its own range, and all other settings matched the primary network. The same-day subsample (N = 116) was used only to describe the distribution of assessment intervals, and no separate network was estimated. The three time windows are nested within one another and were therefore compared descriptively, without network comparison tests.

The sensitivity of the latent profile structure was evaluated through an indicator-resolution sensitivity analysis. In this analysis, the 13 psychometric indicators were replaced by three scale-level scores, namely the HAMA total score, the HAMD-24 total score, and the reverse-scored DBAS-16 mean. These were combined with the 5 PSG indicators to give 8 indicators in total. The model specification, the covariance parameterization comparison, and the model-selection rules matched those of the primary latent profile analysis, including the restriction of selection to the diagonal family, and the resulting classification was compared with the primary classification using a cross-tabulation and the adjusted Rand index. This analysis evaluates the stability of the profile patterns under a change in indicator resolution and is not an external validation or a strictly balanced indicator model.

## 3 Results

### 3.1 Participant characteristics, temporal alignment, and measurement properties

The final sample included 318 patients (201 female, 63.2%) with a mean age of 59.2 ± 12.4 years (range 19–84). PSG recordings were obtained between January 2024 and April 2026. The sample covered the full spectrum of obstructive sleep apnea severity: 85 patients (26.7%) had a normal AHI (<5 events/h), 105 (33.0%) had mild, 79 (24.8%) had moderate, and 49 (15.4%) had severe OSA. The raw AHI distribution contained two exact zero values (0.6%); the remaining 316 values were positive, with a minimum positive value of 0.1 events/h. The median AHI was 10.75 events/h (IQR, 4.70–21.90), and skewness decreased from 1.74 before transformation to −0.28 after the log(AHI + 1) transformation.

The interval between psychometric assessment and PSG was defined as the largest absolute number of days between the PSG date and the dates of the three questionnaires. All 318 patients completed the questionnaires within 7 days of PSG. The median interval was 1 day (interquartile range 0 to 1 day; range 0 to 7 days). Of these, 116 (36.5%) were assessed on the same day, 258 (81.1%) within 1 day, and 312 (98.1%) within 3 days (**Supplementary Tables 4A, B**).

Internal consistency was acceptable for all three questionnaires in the present sample. Ordinal McDonald’s ω was 0.883 (95% CI, 0.855–0.907) for the HAMA-14, 0.879 (0.853–0.901) for the HAMD-24, and 0.896 (0.870–0.917) for the reverse-scored DBAS-16 mean score. Coefficients at the factor level ranged from 0.513 to 0.832, with sleep expectations (two items, Spearman–Brown = 0.513), retardation (ω = 0.590), and medication beliefs (ω = 0.653) showing comparatively lower values. The HAMD weight and diurnal variation factors each consist of a single item, so internal consistency was not calculated. Full descriptive statistics, empirical distributions, and reliability point estimates with 95% CIs for all nodes are reported in **Supplementary Tables 1A**-**3**, **Supplementary Figure 1**.

LPA identified five latent profiles (detailed in Section 3.2). **Table 1** presents participant characteristics stratified by profile. Because the 13 psychometric indicators and 5 PSG indicators were used to define the profiles, group comparisons on these variables would not provide independent evidence of profile differences, and they are therefore reported descriptively only. Formal inference was restricted to the three variables not entered into the LPA. Age differed across profiles, *F*(4, 313) = 4.97, *p* <.001, partial η² = .060. C3 had the lowest mean age (49.2 ± 14.5 years) and was younger than each of the other four profiles after Tukey adjustment (*p* = .007, <.001,.015, and <.001, respectively). Total sleep time also differed; because the Brown-Forsythe test indicated heterogeneity of variance (*p* = .008), Welch ANOVA was used, *F*(4, 72.35) = 4.63, *p* = .002, partial η² = .062. In Games-Howell comparisons, C2 averaged 53.4 minutes shorter than C1 (*p* = .016) and 43.9 minutes shorter than C5 (*p* = .004); no other pairwise comparison reached significance. Sex distribution was associated with profile membership, χ²(4, *N* = 318) = 12.28, *p* = .015, Cramér’s V = .196. The proportion of male patients was highest in C4 (70.0%), compared with 36.8% in the full sample.

**Table 1:** Sample characteristics and profile description for the five-class solution (N = 318).

Characteristic	Overall (N = 318)	C1 (n = 40)	C2 (n = 115)	C3 (n = 26)	C4 (n = 20)	C5 (n = 117)	p
Variables not entered into the latent profile analysis							
Age, years	59.25 (12.35)	59.55 (11.72)	60.07 (11.89)	49.19 (14.49)	60.50 (13.48)	60.36 (11.49)	<.001
Female sex, n (%)	201 (63.2)	23 (57.5)	73 (63.5)	18 (69.2)	6 (30.0)	81 (69.2)	.015
Male sex, n (%)	117 (36.8)	17 (42.5)	42 (36.5)	8 (30.8)	14 (70.0)	36 (30.8)	
Total sleep time, min	365.82 (99.00)	391.49 (85.67)	338.06 (103.88)	398.94 (122.10)	336.40 (119.79)	381.99 (80.60)	.002
Scale-level scores							
HAMA-14 total	15.21 (7.40)	8.07 (4.38)	18.29 (4.45)	30.50 (7.15)	7.95 (3.27)	12.48 (3.86)	
HAMD-24 total	16.25 (7.16)	9.22 (4.06)	20.76 (3.93)	29.31 (6.37)	10.35 (4.08)	12.32 (3.34)	
DBAS-16 mean	3.37 (0.50)	2.50 (0.36)	3.59 (0.40)	3.50 (0.54)	3.40 (0.35)	3.43 (0.29)	
Psychometric indicators entered into the latent profile analysis							
Psychic anxiety	9.12 (4.30)	4.70 (2.58)	10.88 (2.74)	17.65 (3.22)	4.85 (2.46)	7.73 (2.56)	
Somatic anxiety	6.10 (3.78)	3.38 (2.60)	7.41 (2.68)	12.85 (5.06)	3.10 (1.71)	4.75 (2.43)	
Anxiety/somatization	5.08 (2.06)	3.35 (1.81)	5.86 (1.46)	8.27 (2.27)	3.40 (1.31)	4.48 (1.50)	
Weight	0.36 (0.66)	0.07 (0.27)	0.57 (0.75)	0.50 (0.81)	0.25 (0.55)	0.24 (0.57)	
Cognitive impairment	2.28 (2.08)	0.85 (1.23)	3.13 (1.55)	6.12 (2.76)	1.10 (1.12)	1.29 (1.08)	
Diurnal variation	0.46 (0.60)	0.32 (0.62)	0.76 (0.57)	0.92 (0.74)	0.25 (0.44)	0.14 (0.35)	
Retardation	2.17 (1.76)	0.72 (1.04)	3.05 (1.42)	4.88 (1.77)	1.50 (1.28)	1.31 (1.08)	
Sleep disturbance	3.82 (1.45)	2.90 (1.81)	4.19 (1.30)	4.12 (1.03)	3.05 (1.85)	3.83 (1.29)	
Hopelessness	2.08 (1.85)	1.00 (1.22)	3.19 (1.64)	4.50 (1.75)	0.80 (0.89)	1.04 (1.04)	
Consequences of insomnia	3.42 (0.66)	2.34 (0.44)	3.69 (0.49)	3.64 (0.67)	3.44 (0.57)	3.46 (0.49)	
Worry about sleep	3.56 (0.54)	2.67 (0.48)	3.74 (0.43)	3.65 (0.59)	3.49 (0.43)	3.68 (0.33)	
Sleep expectations	3.24 (0.80)	2.77 (0.84)	3.35 (0.81)	3.62 (0.93)	3.40 (0.70)	3.17 (0.69)	
Medication beliefs	3.01 (0.66)	2.23 (0.44)	3.27 (0.65)	2.87 (0.79)	3.13 (0.45)	3.03 (0.51)	
PSG indicators entered into the latent profile analysis							
Sleep efficiency, %	68.30 (15.65)	74.71 (11.60)	63.58 (17.20)	71.25 (16.59)	58.83 (17.29)	71.72 (12.63)	
log(AHI + 1)	2.39 (1.03)	2.38 (0.97)	2.41 (0.98)	1.93 (1.22)	3.89 (0.60)	2.22 (0.89)	
Arousal index, events/h	25.83 (14.20)	22.57 (10.13)	26.15 (11.66)	22.02 (13.00)	60.82 (16.33)	21.48 (8.08)	
N3 sleep, %	10.18 (9.54)	12.98 (10.93)	11.29 (10.45)	9.30 (9.26)	2.06 (3.89)	9.72 (8.04)	
REM sleep, %	16.98 (7.76)	19.91 (6.70)	15.69 (8.42)	16.75 (9.60)	12.46 (8.25)	18.07 (6.29)	

Mean (SD) for continuous variables, n (%) for categorical variables. Formal inference is restricted to age, sex, and total sleep time, the only variables not entered into the latent profile analysis; age and total sleep time were compared with one-way ANOVA and Tukey-adjusted pairwise tests, sex with a Pearson chi-square test. All other variables defined the profiles and are reported descriptively only. DBAS-16 scores are reverse scored; AHI is on the log(AHI + 1) scale.

### 3.2 Latent profile analysis

**Table 2** presents fit indices for the selected parameterization across one to six profiles. Of the 84 models fitted, 51 converged with non-singular covariance estimates; VVI and EVI, the two most flexible diagonal parameterizations, returned no fit for any solution with more than one profile. Sixteen diagonal-family models met the prespecified eligibility criteria, and among these the VEI specification with five profiles had the lowest BIC (15,325.7), an entropy of 0.897, and a smallest class comprising 6.3% of the sample. It was therefore retained. The corresponding six-profile VEI solution had a lower AIC but a higher BIC, and its smallest class contained 13 patients (4.1%), which did not meet the minimum class size criterion. Two ellipsoidal models reached lower BIC values (EEE with two and three profiles, 15,241.1 and 15,291.1) but fall outside the model family appropriate to latent profile analysis and were not eligible for selection (Section 2.4.2). The full comparison is reported in **Supplementary Table 10**. Mean posterior probabilities in the selected model ranged from 0.913 to 0.980 across profiles (**Supplementary Tables 5A, B**). The latent profile analysis used all 18 indicators. One indicator was subsequently removed from the network node set (see Section 3.3), which affects the network analyses only and does not alter the profile structure reported here.

**Table 2:** Latent profile analysis fit indices for one- to six-class solutions.

Classes	logLik	AIC	BIC	aBIC	Entropy	Smallest class, %	Class sizes, n (%)
1	−8112.99	16,297.98	16,433.41	16,319.23	1.000	100.0	318 (100.0)
2	−7709.63	15,531.25	15,741.93	15,564.31	0.862	39.0	194 (61.0), 124 (39.0)
3	−7533.40	15,218.80	15,504.72	15,263.66	0.878	17.0	54 (17.0), 101 (31.8), 163 (51.3)
4	−7423.52	15,039.03	15,400.19	15,095.69	0.898	8.8	47 (14.8), 108 (34.0), 28 (8.8), 135 (42.5)
**5^a^**	**−7328.63**	**14,889.26**	**15,325.66**	**14,957.73**	**0.897**	**6.3**	**40 (12.6), 115 (36.2), 26 (8.2), 20 (6.3), 117 (36.8)**
6^b^	−7279.17	14,830.33	15,341.97	14,910.61	0.894	4.1	49 (15.4), 105 (33.0), 24 (7.5), 33 (10.4), 94 (29.6), 13 (4.1)

All 14 covariance parameterizations available in mclust were fitted for one to six profiles. Selection was restricted to the six parameterizations with diagonal covariance matrices, consistent with the local independence assumption of latent profile analysis, and required entropy of at least 0.80 and a smallest class of at least 5% of the sample, applied together. VVI and EVI returned no fit for any solution with more than one profile. The five-class VEI solution met both criteria and had the lowest BIC among eligible diagonal models; the six-class VEI solution was ineligible because its smallest class contained 4.1% of the sample. Two ellipsoidal models reached lower BIC values but fall outside the diagonal family and were not eligible for selection. The complete comparison of all 84 fitted models is reported in **Supplementary Table 10**. logLik, log-likelihood; aBIC, sample-size-adjusted BIC.

^a^Retained solution: lowest BIC among eligible diagonal models, entropy 0.897, smallest class 6.3%. ^b^Ineligible: smallest class contained 13 patients (4.1%), below the 5% criterion, despite a lower AIC.

Bold values indicate the retained solution.

**Figure 1:**
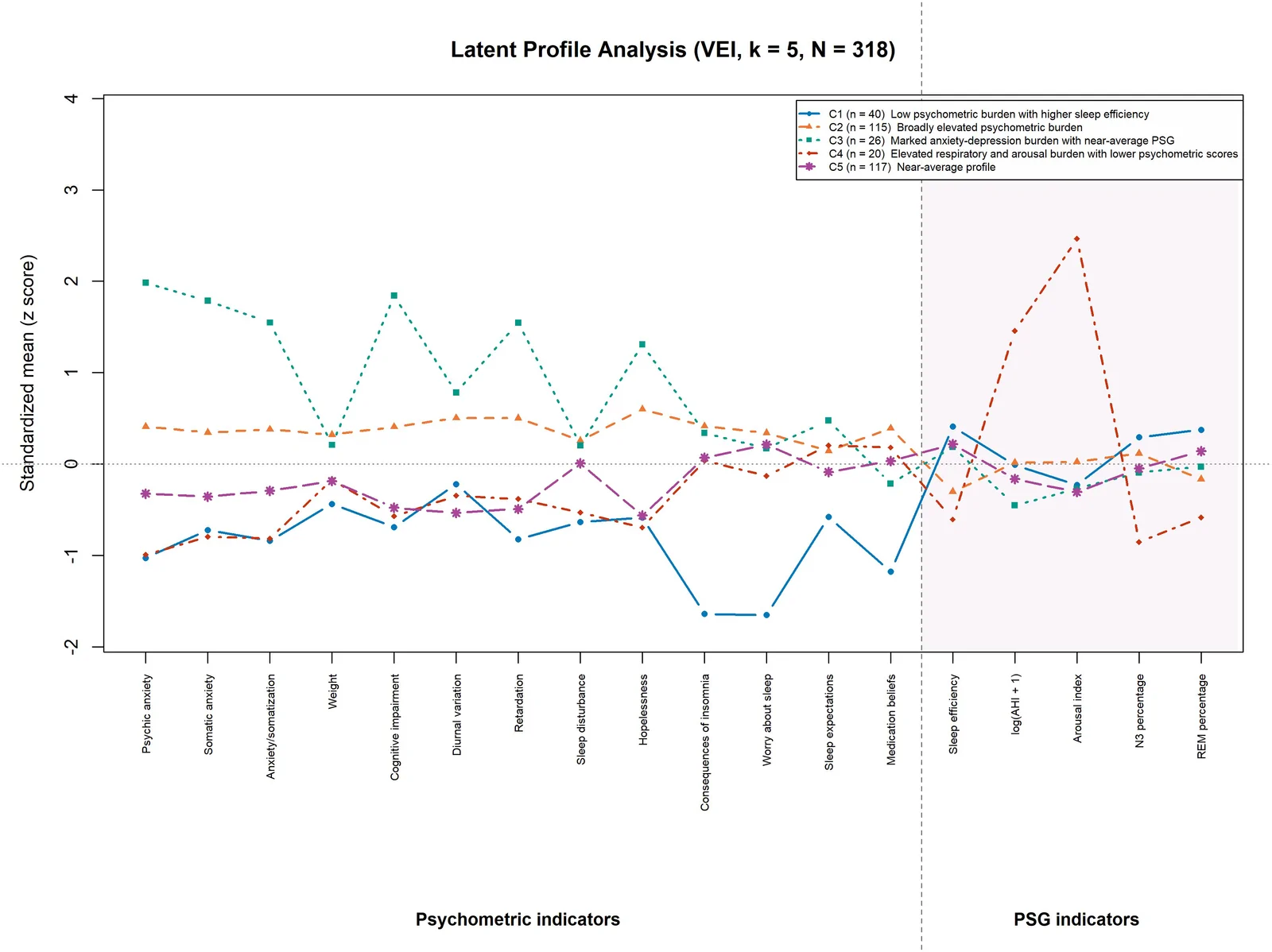
Standardized indicator means for the five latent profiles (N = 318). Z scores across the full sample for all 18 latent profile indicators. The dashed line and shaded panel separate the two modalities. DBAS-16 items were reverse scored, so higher values on the 13 psychometric indicators indicate greater severity or more dysfunctional beliefs. PSG values have no uniform direction: higher sleep efficiency and N3 percentage are generally favorable, whereas higher log(AHI + 1) and arousal index are not. Anxiety/somatization was later removed from the network node set and does not appear in **Figures 2** and **3**.

**Figure 1** displays standardized mean scores across the 18 indicators for each profile. The profile labels summarize the indicator patterns observed in the present sample and should not be taken as validated clinical subtypes. C1 (n = 40, 12.6%; low psychometric burden with higher sleep efficiency) scored below the sample mean on all 13 psychometric indicators, with consequences of insomnia and worry about sleep particularly low (Z = −1.64 and −1.65), while sleep efficiency and REM percentage were above the mean (Z = +0.41 and +0.38). C2 (n = 115, 36.2%; broadly elevated psychometric burden) showed moderate elevations across most anxiety, depression, and sleep belief indicators, with sleep efficiency and REM percentage slightly below the mean. C3 (n = 26, 8.2%; marked anxiety-depression burden with near-average PSG) showed marked elevations on the anxiety and depression indicators, reaching Z = +1.98 for psychic anxiety, +1.84 for cognitive impairment, and +1.79 for somatic anxiety, while all five PSG indicators remained close to the sample mean (Z from −0.45 to +0.19). C4 (n = 20, 6.3%; elevated respiratory and arousal burden with lower psychometric scores) showed the opposite pattern: arousal index and log(AHI + 1) were clearly elevated (Z = +2.47 and +1.46), sleep efficiency, N3 percentage, and REM percentage were below the mean (Z = −0.61, −0.85, and −0.58), and all nine HAMA and HAMD factors fell below the sample mean, while the four DBAS factors remained close to it (Z from −0.13 to +0.21). C5 (n = 117, 36.8%; near-average profile) was the largest profile, with all indicators within 0.6 SD of the sample mean.

C3 and C4 differed in opposite directions across the two indicator sets. C3 combined elevated psychometric scores with near-average PSG values, whereas C4 combined clearly abnormal PSG values with lower psychometric scores. This contrast describes the profile mean patterns in the present sample. Both profiles were small (26 and 20 patients), and their indicator patterns and labels may be sensitive to sampling variation and to the composition of this single-center sample. They should therefore be regarded as exploratory.

### 3.3 Gaussian graphical model

Before estimating the final network, we screened the 78 pairwise combinations of the 13 psychometric indicators for redundancy using the Goldbricker procedure (p = .05, hittner2003 method, threshold = .25, minimum correlation = .50). Nine pairs met the candidate criteria, five of which involved the HAMD anxiety/somatization factor. This factor overlapped with both psychic anxiety and somatic anxiety, two HAMA factors with more clearly defined content, and its internal consistency (ω = 0.595) was lower than that of either factor (0.821 and 0.802). It was therefore removed. The remaining candidate pairs consisted of non-overlapping items belonging to separately defined content domains and were all retained (**Supplementary Table 7**). The node set was fixed at 17 indicators, and the final network was then estimated.

The EBICglasso estimation retained 43 non-zero edges out of 136 possible (density 0.316), of which 37 were positive and 6 were negative. The three strongest edges were consequences of insomnia–worry about sleep (0.483), log(AHI + 1)–arousal index (0.471), and psychic anxiety–somatic anxiety (0.392), all connecting nodes within the same measurement modality. Three edges connected psychometric indicators to PSG indicators. All three were negative, with a mean weight of −0.027: psychic anxiety–log(AHI + 1) (−0.038), medication beliefs–REM percentage (−0.034), and sleep disturbance–sleep efficiency (−0.010).

**Figure 2:**
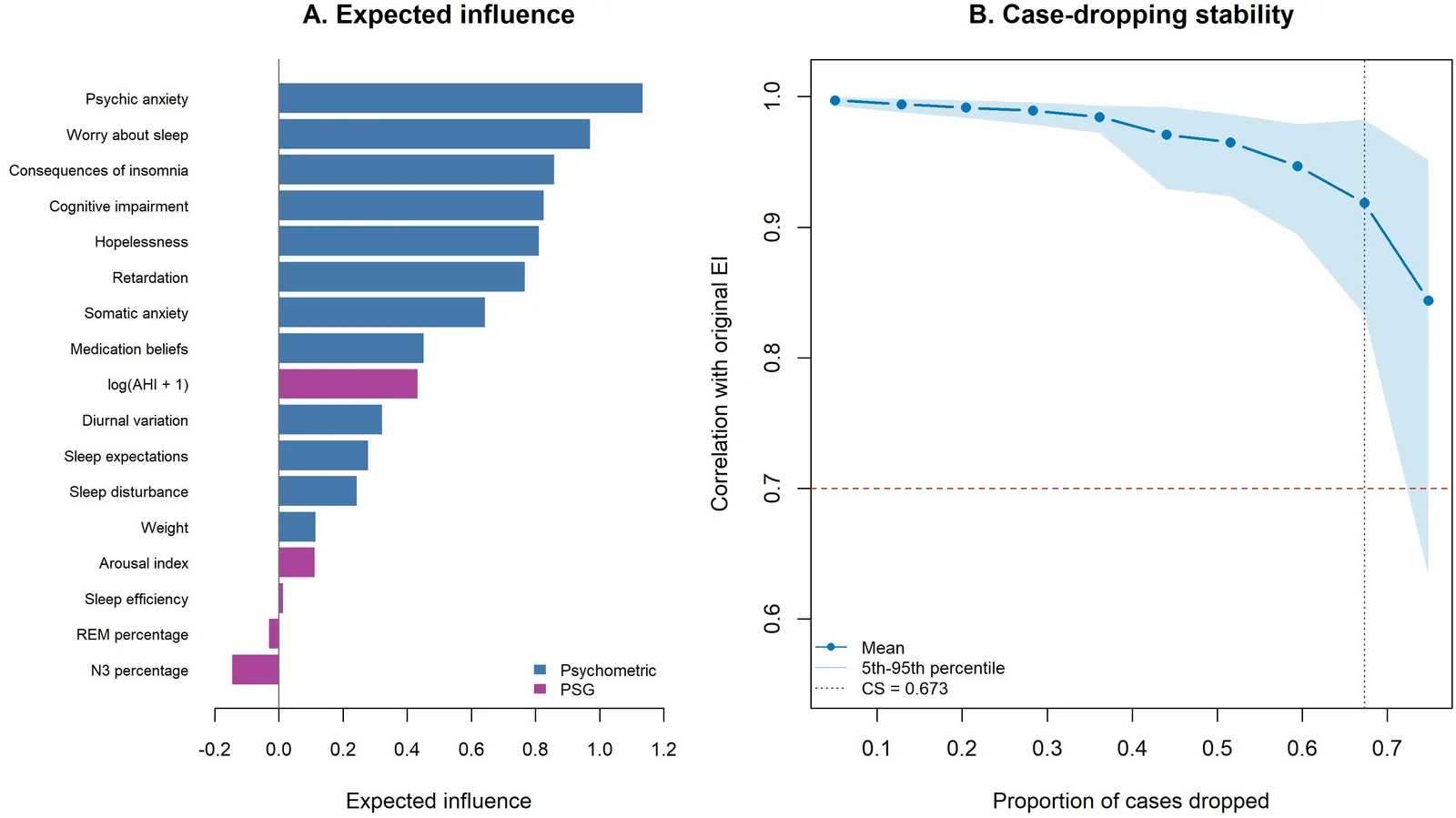
Expected influence and case-dropping stability for the 17-node network. **(A)** Expected influence for the 17 nodes, ordered by magnitude and colored by modality. Expected influence is the sum of signed edge weights at a node and describes relative connectivity within this network, not clinical importance. **(B)** Case-dropping bootstrap: mean correlation with the original ordering, the 5th to 95th percentile band, the 0.70 reference line, and an expected-influence correlation-stability coefficient of.673.

**Figure 3:**
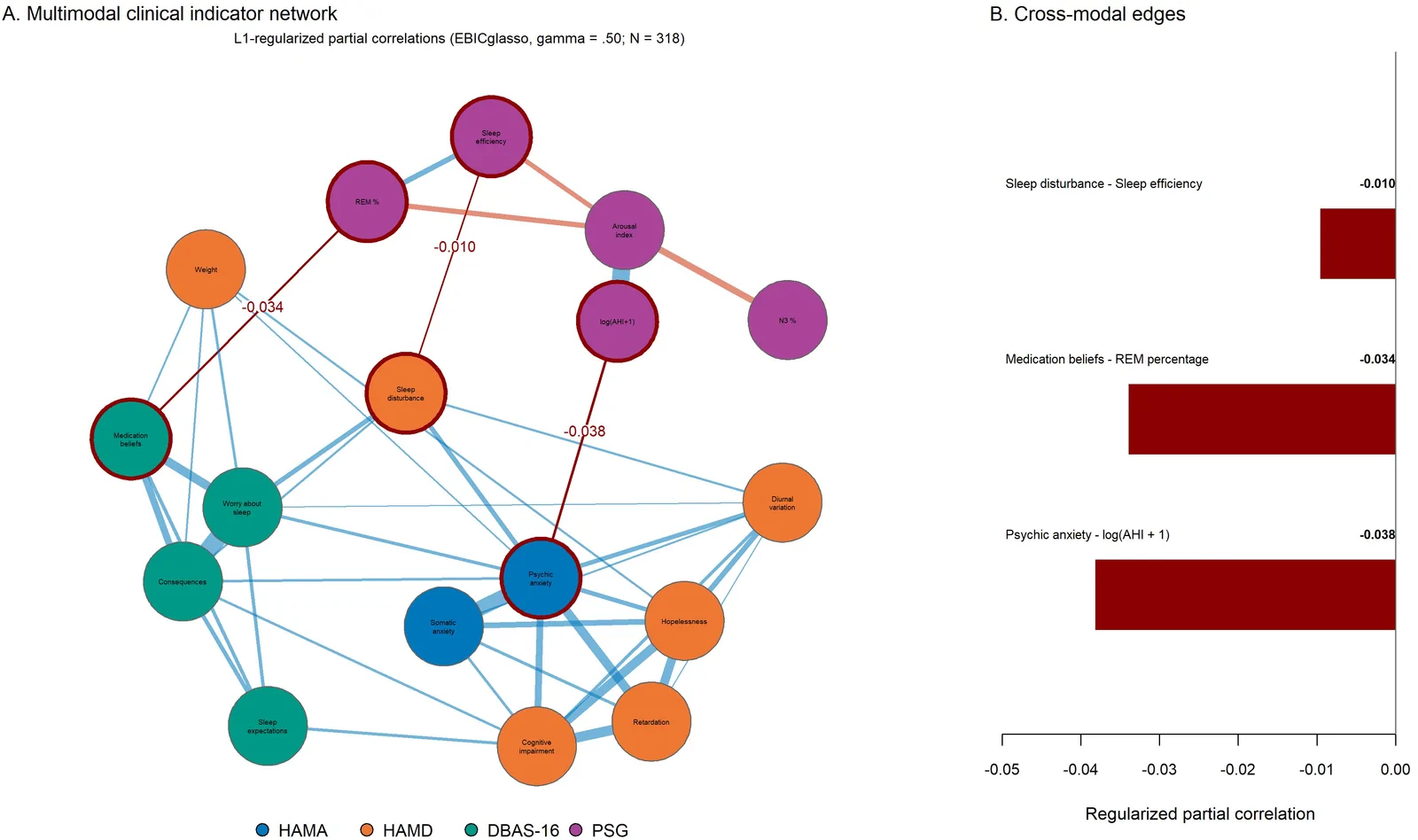
Final 17-node multimodal clinical indicator network and its cross-modal edges (N = 318). **(A)** Network estimated with EBICglasso (gamma = 0.50) on a cor_auto correlation matrix, Fruchterman-Reingold layout. Node color denotes instrument; blue edges are positive and red negative, with thickness proportional to weight. Three of the 43 non-zero edges are cross-modal; these are drawn in solid dark red with weights labeled, and the six nodes they connect are outlined in dark red. **(B)** The same three edges on a common scale. Each non-zero bridge expected influence equals the single cross-modal edge weight of that node; the other 11 nodes have a value of zero. All three intervals include zero (**Supplementary Figure 2**).

**Figure 2** presents expected influence (EI) for the 17 nodes (**Figure 2A**) and the case-dropping stability of expected influence (**Figure 2B**). Psychic anxiety had the highest EI (1.134), followed by worry about sleep (0.970), consequences of insomnia (0.858), cognitive impairment (0.825), and hopelessness (0.811). Log(AHI + 1) had an EI of 0.433 and ranked ninth. The remaining four PSG indicators occupied the four lowest ranks, with negative values for REM percentage (−0.031) and N3 percentage (−0.144). **Figure 3A** displays the estimated network, and **Figure 3B** presents the three cross-modal edges and the corresponding bridge expected influence (bEI) values. Among the psychometric indicators, psychic anxiety had the largest absolute value (−0.038), followed by medication beliefs (−0.034) and sleep disturbance (−0.010). Among the PSG indicators, the corresponding values were log(AHI + 1) (−0.038), REM percentage (−0.034), and sleep efficiency (−0.010). Because the network retained only three cross-modal edges, each non-zero bEI value equals the weight of the single cross-modal edge connected to that node, and the remaining 11 nodes had a bEI of zero. Bootstrap intervals for all three cross-modal edges included zero.

A nonparametric bootstrap of the edge weights (1,000 resamples, participants sampled with replacement) yielded 95% percentile intervals whose width, across all 136 possible edges, had a median of 0.053 and a maximum of 0.257. Of the 43 non-zero edges, 19 had intervals that did not cross zero (**Supplementary Table 9**, **Supplementary Figure 2**). These intervals describe estimation precision; they were not used to determine whether an edge is present, and they do not constitute a formal test of differences between edge weights. Case-dropping bootstrap of expected influence used 1,000 resamples across ten levels of case removal ranging from 5% to 75%. The correlation stability coefficient was.673 (**Figure 2B**), indicating that after dropping approximately 67% of cases, more than 95% of the resampled EI vectors retained a correlation of at least 0.70 with the original estimates. This coefficient describes the stability of expected influence under case removal and does not indicate that all individual edges or the network as a whole are equally stable. Data-driven community detection identified three communities in the primary network rather than the two prespecified modalities: the 5 PSG indicators formed one community; the 4 sleep belief factors together with HAMD weight and sleep disturbance formed a second; and the 2 HAMA factors together with the remaining 4 HAMD factors formed a third. Sleep disturbance therefore clustered with the sleep belief factors rather than with the other HAMD factors. Normalized mutual information between this partition and the prespecified two-modality grouping was 0.712. The Spinglass algorithm identified four communities, differing in that it further subdivided the PSG indicators, with a normalized mutual information of 0.638.

### 3.4 Network comparison test and moderated network analysis

The network comparison test compared the two largest profiles, C5 (n = 117) and C2 (n = 115). Both networks were estimated with gamma = 0, and the test used 1,000 permutations. Global strength was 0.967 in C5 and 1.271 in C2, and the difference was not significant (S = 0.304, p = .819). The test of network structure invariance was also not significant (M = 0.187, p = .808). None of the 136 edges differed between the two networks after BH correction, and none of the 17 nodes differed in expected influence. Because the five profiles differ along several dimensions and do not form a single ordering from lower to higher psychometric burden, no pooled group comparison was conducted.

The moderated network analysis used the full sample (N = 318) and included the 16 nodes of the final node set other than AHI, with the standardized log(AHI + 1) score as a continuous moderator. The model was fitted with mgm using 10-fold cross-validation to select the regularization parameter and the AND rule. No third-order interaction parameters were retained. The conditional networks obtained at −1 SD, the mean, and +1 SD of the moderator were identical, each containing 24 non-zero edges with a global strength of 4.250 and a maximum absolute edge weight of 0.543. The largest absolute difference in edge weights between the low and high AHI conditions was zero.

### 3.5 Sensitivity analyses

We examined the sensitivity of the primary network to the estimation settings in three ways: removing the two HAMD factors with pronounced floor effects (weight and diurnal variation) while returning the anxiety/somatization factor that had been removed for content overlap, which produced a 16-node network; lowering the EBIC model-selection hyperparameter from 0.50 to 0; and using Spearman correlations with the ranks of the untransformed AHI. These networks retained 44, 55, and 54 edges, respectively. Each contained all non-zero edges of the primary network across the shared nodes, none were lost, and sign agreement was 100%. Correlations between the full edge weight vectors and those of the primary network ranged from.977 to.995, and rank correlations for expected influence ranged from.966 to 1.000. The three networks contained 8, 11, and 10 cross-modal edges, compared with 3 in the primary network, and rank correlations for bridge expected influence among the PSG indicators ranged from.564 to.872. Full comparisons across all seven prespecified metrics are reported in **Supplementary Tables 8A-D**, and the four networks are shown under a unified layout and edge scale in **Supplementary Figure 4**.

Network structure remained stable when the interval between psychometric assessment and PSG was narrowed. The ≤3-day subsample (N = 312) retained 42 edges and the ≤1-day subsample (N = 258) retained 44 edges, each estimated after restandardizing within the respective subsample. Relative to the primary network, both retained 41 edges and lost 2, with the lost edges having weights no greater than 0.021. Edge weight correlations were.999 and.991, and sign agreement was 100% in both cases. The five highest-ranking nodes on expected influence were identical across the three networks, and all three cross-modal edges of the primary network were retained under both narrower windows. The ≤1-day network additionally contained one further cross-modal edge between the HAMD weight factor and sleep efficiency (−0.028), and the rank correlation for bridge expected influence among the PSG indicators fell to.579 in this subsample, indicating that bridge rankings are more sensitive to the matching window than the overall network structure.

To assess the influence of the unequal numbers of psychometric and PSG indicators, we repeated the latent profile analysis using eight indicators: the HAMA total score, the HAMD-24 total score, the reverse-scored DBAS-16 mean, and the 5 PSG indicators. The same parameterization comparison and selection rules were applied, and the EVI specification with five classes was retained (**Supplementary Tables 6A-C**, **Supplementary Figure 3**). The adjusted Rand index between the two classifications was 0.264, indicating partial agreement. Agreement varied systematically across profiles. Of the 20 patients in C4, which is defined mainly by its PSG values, 18 (90.0%) fell within a single sensitivity class. Agreement was lower for the profiles defined mainly by psychometric patterns: the corresponding proportions were 65.4% for C3 and 52.2% for C2. This pattern indicates that separation along the respiratory dimension does not depend on indicator resolution, whereas the psychometric profiles do.

## 4 Discussion

This study integrated PSG parameters alongside psychometric factor scores in a single Gaussian graphical model, an approach that, to our knowledge, has not been previously reported in sleep-network research. The estimated network was dominated by within-modality associations, with few and weak cross-modal conditional associations: only 3 of the 136 possible pairwise associations were retained as cross-modal edges, all of them negative, with a mean weight of −0.027, and four of the five PSG indicators occupied the four lowest ranks on signed expected influence. For bridge expected influence, psychic anxiety had the largest absolute value among the psychometric indicators (−0.038) and log(AHI + 1) among the PSG indicators (−0.038), followed by medication beliefs (−0.034). Latent profile analysis identified five profiles, two of which were small and showed opposite patterns: C3 combined markedly elevated anxiety and depression indicators with near-average PSG values, whereas C4 combined clearly abnormal respiratory and arousal indicators with lower psychometric scores. The network comparison test detected no statistically significant differences between the two largest profiles, and the moderated network analysis retained no nonzero moderation parameters involving AHI. These findings describe the cross-modal conditional association structure observed in the present sample and should not be interpreted as evidence of system-level decoupling, causal direction, or mechanistic independence between psychological and physiological processes.

### 4.1 Interpretation of cross-modal conditional associations

The estimated network contained three small nonzero cross-modal edges, all of which were negative (mean weight = −0.027). Four of the five PSG indicators also showed comparatively low signed Expected Influence values.

Subjective-objective sleep discrepancy is well recognized in sleep medicine (9). Meta-analytic evidence shows that even when insomnia patients exhibit measurable PSG abnormalities, the effect sizes are small relative to the severity of their subjective complaints (8). However, prior studies relied on bivariate correlations or group comparisons, leaving open the possibility that confounding variables account for the observed discrepancies. Our GGM estimated partial correlations controlling for all other nodes simultaneously. The negative cross-modal edges observed in this multivariable model represent conditional associations and do not establish that unmeasured confounding has been excluded or that the two measurement modalities are structurally separated. Although within-psychometric, within-PSG, and cross-modal edges share the same statistical definition as undirected conditional associations, their substantive interpretations are not equivalent; cross-modal edges should not be interpreted as interactions between interchangeable symptoms or as physiological-to-psychological transmission.

An important alternative interpretation concerns measurement unreliability and temporal mismatch across modalities. The psychometric indicators reflect broader clinical states or symptom periods, whereas the PSG indicators were derived from one selected laboratory night. Hu et al. (31) reported first-night effects affecting sleep continuity and aspects of sleep architecture in patients with insomnia disorder. Newell et al. (32) likewise observed first-night effects across several sleep-laboratory populations and clinically relevant night-to-night variability in AHI among patients with sleep-related breathing disorders. First-night effects, night-to-night variability, differences in temporal coverage, sampling uncertainty, and regularization may attenuate or otherwise alter the estimated cross-modal conditional associations. Because the study did not include a standardized repeated-PSG protocol, we could not estimate within-person PSG reliability, quantify the contribution of night-specific variability, or correct the network estimates for measurement error. Narrowing the matching window between the psychometric assessment and PSG does not resolve this difference in temporal reference, because the psychometric instruments refer to periods of one week or longer whereas PSG captures a single night. Although these measurement limitations provide a plausible explanation for small-magnitude associations, the present data cannot determine whether or how they contributed to the uniformly negative signs of the retained cross-modal edges. The direction and magnitude of these edges should therefore be interpreted descriptively.

In partial correlation terms, higher values on some psychometric indicators were associated with lower or more favorable values on selected PSG parameters after accounting for the other included indicators. Harvey’s cognitive model of insomnia (6) provides one possible framework for understanding why subjective distress may not closely correspond to single-night PSG findings; however, the present cross-sectional analysis cannot test this proposed mechanism. A related pattern appears in the relative connectivity of the two modalities. In the present network, psychic anxiety had the highest Expected Influence (1.134), whereas four of the five PSG indicators occupied the four lowest ranks. This pattern reflects relative connectivity within the estimated network and does not demonstrate that psychological processes causally maintain distress. Clinically, normal PSG results should not be used to dismiss a patient’s subjective experience of poor sleep.

### 4.2 Cross-modal connectivity of psychic anxiety, medication beliefs, and sleep efficiency

The three psychometric indicators connected by cross-modal edges occupy very different positions within the network. Psychic anxiety ranked first in expected influence among the 17 nodes (1.134), medication beliefs ranked eighth (0.452), and sleep disturbance ranked twelfth (0.243). Conversely, the six nodes ranked second through seventh, namely worry about sleep, consequences of insomnia, cognitive impairment, hopelessness, retardation, and somatic anxiety, had no non-zero connection with any PSG indicator. Occupying a central position within the psychometric modality and having a cross-modal connection are therefore two different things. The most densely connected psychometric indicators were strongly associated with one another and formed an internally cohesive cluster, yet this cluster as a whole showed almost no conditional association with the PSG indicators.

The negative conditional association between psychic anxiety and log(AHI + 1) runs in the same direction as the pattern observed at the profile level. C3 was characterized by markedly elevated anxiety and depression indicators alongside near-average PSG values, whereas C4 showed clearly abnormal respiratory and arousal indicators alongside lower psychometric scores. The negative edge at the variable level and the two-directional contrast at the group level come from different analytic approaches yet point to the same separation. One possible interpretation is that respiratory-predominant and distress-predominant presentations correspond to different referral pathways in a sleep center population. Patients in the former group often present because of snoring, witnessed apneas, or daytime sleepiness rather than because of subjective distress, whereas patients in the latter group report substantial distress with respiratory indices that may fall within the normal range. Bjorvatn et al. (10) reported an association in the same direction at the bivariate level, providing external reference for this pattern. C3 and C4 comprised 25 and 22 patients respectively, so this contrast is descriptive.

The negative edge between medication beliefs and REM percentage admits an alternative explanation that the present study cannot rule out. Benzodiazepines alter sleep architecture, including a reduction in REM sleep time (33), and patients scoring higher on medication beliefs are also more likely to be using hypnotic medication. If so, this edge may reflect actual medication use affecting both the belief score and REM percentage, rather than a direct association between a cognitive factor and sleep architecture. This interpretation also offers a perspective on two earlier findings. Chung et al. (17) reported that medication beliefs was the only DBAS-16 factor that did not improve after CBT-I, and Morin et al. (14) found that the DBAS total score was unrelated to any PSG parameter. If medication beliefs is partly anchored in actual medication use rather than in cognitive bias alone, its relative insensitivity to cognitive intervention becomes easier to understand, and because the remaining three DBAS factors are purely cognitive in content, the total score would dilute this specific association. Song et al. (34) found that changes in individual subscales after CBT-I predicted different clinical outcomes, which likewise supports modeling at the factor level rather than the total-score level. The present study did not collect medication records and cannot test this explanation; including medication information in future studies would allow it to be distinguished.

Notably, the cross-modal edge whose direction is most consistent with clinical intuition, that between sleep disturbance and sleep efficiency, was the weakest of the three (−0.010). Subjective reports of disturbed sleep and lower objective sleep efficiency would be expected to correspond, yet after accounting for the remaining indicators their conditional association was close to zero. The network position of the sleep disturbance factor points to a reason: it ranked twelfth in expected influence and, in the data-driven partition, clustered with the sleep belief factors rather than with the other HAMD factors, suggesting that it captures subjective appraisal of sleep more than the somatic components of depression. Trimmel et al. (35) reported that insomnia patients underestimate their own sleep efficiency by a median of 11%. If subjective appraisal departs systematically from objective values, the weakness of this edge need not be read as evidence that the two are unrelated, but rather as an indication that subjective appraisal and objective parameters do not map onto one another on a one-to-one basis. All of these interpretations are descriptive, and the present cross-sectional design cannot establish any of them.

### 4.3 Descriptive characteristics of the latent profiles

The overall structure of the five profiles indicates that the PSG indicators distinguish only one of them. Apart from C4, the standardized means of the five PSG indicators in the remaining four profiles fell entirely within ±0.50, whereas the psychometric indicators in these same four profiles spanned a much wider range, from −1.65 for worry about sleep in C1 to +1.98 for psychic anxiety in C3. The four profiles that together comprise 93.7% of the sample are therefore separated mainly by the level of the psychometric indicators, while their sleep physiology is comparatively similar. Only C4, which comprises 6.3% of the sample, departed clearly from this pattern, with an arousal index of +2.47 and a log(AHI + 1) value of +1.46.

This pattern runs in the same direction as the network results. In the 17-node network, the eight nodes with the highest expected influence were all psychometric indicators, and four of the five PSG indicators occupied the four lowest ranks. The classification analysis and the network analysis address different questions, but both indicate that the information distinguishing individuals in this sample lies mainly on the psychometric side. This reflects how the two sets of indicators varied in the present sample rather than any difference in their clinical importance.

C3 and C4 were both small profiles, and they did not prove equally robust. In the indicator-resolution sensitivity analysis, 18 of the 20 patients in C4 (90.0%), a profile defined mainly by its PSG values, fell within a single sensitivity class, whereas the corresponding proportions for the profiles defined mainly by psychometric patterns were lower, 65.4% for C3 and 52.2% for C2; the adjusted Rand index between the two classifications was 0.264. Profiles defined primarily by PSG features thus appear relatively insensitive to a change in the resolution of the psychometric indicators, whereas profiles defined primarily by psychometric features depend on factor-level differentiation. C4 also had a higher proportion of male patients (70.0%) than the sample as a whole (36.8%), consistent with the higher prevalence of obstructive sleep apnea in men reported by Benjafield et al. (1). Because these two profiles contained only 26 and 20 patients, and because the detection of the number of profiles is itself subject to statistical power (36), and because no independent validation sample was available, these characteristics should be read descriptively, and the profile labels await examination in larger multicenter samples.

### 4.4 Network comparison and AHI moderation findings

The exploratory NCT did not detect statistically significant network differences between the two largest latent profiles, and the exploratory MNA retained no nonzero moderation parameters involving AHI. The NCT detected no significant differences between C5 and C2 in global strength (p = .819) or network structure (p = .808). This comparison did not assess the stability of the five-profile solution or validate the individual smaller profiles.

These null findings require cautious interpretation. Van Borkulo et al. (29) noted that NCT requires both sufficient sample sizes and substantial between-group differences to achieve adequate power. The absence of statistically significant NCT results or nonzero MNA parameters does not establish invariance or equivalence under the specified models. The absence of a retained nonzero AHI moderation parameter also has several non-mutually exclusive explanations, including a genuinely small or absent moderation effect, limited statistical sensitivity, shrinkage under the regularized model, and variability in a single-night AHI assessment. Because the study did not include a standardized repeated-PSG protocol, these possibilities cannot be distinguished using the present data. The result should therefore not be interpreted as evidence that AHI has no moderating role. The inverse associations between AHI and anxiety and depression reported by Bjorvatn et al. (10) provide relevant external context but do not establish invariance of the conditional network structure.

Luo et al. (13) conducted a network analysis of depression, anxiety, and sleep disturbance symptoms in 621 OSA patients and reported that NCT edge weights differed between mild-to-moderate and severe groups. Their item-level analysis detected differences that our factor-level model did not, suggesting that analytical granularity may influence whether severity-related differences emerge.

### 4.5 Strengths, limitations, and future directions

To our knowledge, this is the first sleep-network study to integrate PSG parameters with psychometric indicators in a single multimodal Gaussian graphical model, allowing within-modality and cross-modal conditional associations to be characterized beyond traditional bivariate analyses. The four-layer analytical framework examined the same dataset from complementary perspectives, with each layer addressing a distinct analytical question. Factor-level modeling allowed domain-specific associations to be examined; whether these associations would be obscured in a total-score model would require a direct comparison of the two approaches. The network results were stable across several specifications: the rank correlation for expected influence was.993 after removing the two factors with floor effects, 1.000 when the EBIC model-selection hyperparameter was lowered to 0, and.966 when Spearman correlations were used. When the interval between psychometric assessment and PSG was narrowed to 3 days and to 1 day, edge weight correlations with the primary network were.999 and.991, and the five highest-ranking nodes on expected influence were identical. In addition, redundancy screening was carried out before the network was estimated, removing one indicator that overlapped substantially with two factors of more clearly defined content, which sharpened the content boundaries of the final node set. This screening applies an operational correlation-pattern criterion; the absence of a flag does not establish that conceptual content overlap has been eliminated, nor does elevated centrality indicate causal, clinical, or intervention importance. Expected influence had a correlation stability coefficient of.673 under case-dropping bootstrap. This coefficient describes the stability of expected influence under case removal only; it does not indicate that all individual edge weights were estimated precisely or that the network structure as a whole was equally stable.

Bridge expected influence requires modality-specific interpretation because the two prespecified groups differed in size: each psychometric indicator had 5 possible PSG neighbors, whereas each PSG indicator had 12 possible psychometric neighbors. Raw bridge expected influence values should therefore not be treated as directly comparable measures of clinical, biological, or etiological importance across modalities.

Several limitations should be noted. First, this was a single-center retrospective study, and the sample was restricted to patients who completed all three questionnaires and had analyzable PSG data. Because the available PSG analytic-source file had already been generated from a list restricted to patients with completed psychometric assessments, we could not reconstruct the number or characteristics of PSG-tested patients without a complete questionnaire set. Complete-record selection may therefore have introduced selection bias, and the findings are most directly generalizable to sleep-center patients able to complete both psychometric assessment and PSG. Second, no *a priori* sample-size calculation or simulation-based power analysis was conducted, and neither the study protocol nor the analysis plan was preregistered. The entropy value and mean posterior probabilities describe classification separation within the selected model; they do not establish that the five-profile solution is the uniquely correct solution, externally reproducible, or clinically valid. In addition, the two most flexible diagonal covariance parameterizations could not be estimated at the present sample size and indicator count, so model selection was necessarily confined to the estimable set; a specification permitting fully profile-specific variances might be preferred in a larger sample. The sample size of 318 was determined by the retrospective records meeting the eligibility criteria rather than by a prospectively specified precision or power target for the 17-node network. Although EBICglasso reduces effective model complexity by shrinking small coefficients, the network still involves 136 possible pairwise associations, and regularization does not eliminate sampling uncertainty, so the precision of weak edges may be limited. As noted above, the null network comparison results and the absence of retained moderation parameters should not be read as evidence of equivalence or invariance. Third, nodes were defined at the factor level rather than the item level, which may have masked connections at the level of individual symptoms; item-level network analyses could provide finer resolution. Several factors also showed limited internal consistency, most notably sleep expectations, retardation, and medication beliefs, which may contribute to measurement error in the corresponding nodes and attenuate their estimated associations. Fourth, the PSG indicators came from a single overnight laboratory recording. First-night effects (31) and night-to-night variability in respiratory events (32) mean that a single night may not represent an individual’s stable sleep physiology, and the study included no standardized repeated-PSG protocol, so within-person reliability could not be estimated and the estimates could not be corrected for measurement error. The implications of this limitation for the cross-modal associations are discussed above. The fixed log(AHI + 1) transformation reduced marginal right skewness but did not establish multivariate normality, and the choice of an additive constant can affect parameter estimates and statistical inference for log-transformed predictors with zero values (37), so the value of 1 should be regarded as a fixed analytic choice. Fifth, a cross-sectional undirected Gaussian graphical model cannot determine the temporal direction or causal origin of the estimated edges, and a conditional association between a psychometric indicator and a PSG indicator may reflect an effect of the psychological state on subsequent sleep physiology, an effect of sleep continuity, architecture, or respiratory disturbance on subsequent psychological symptoms or sleep-related beliefs, reciprocal processes over time, or unmeasured common causes acting on both. Conditioning on the remaining observed indicators and applying L1 regularization do not distinguish among these explanations or eliminate unmeasured confounding. The module control framework proposed by Pan et al. (38) offers a complementary approach for characterizing model-derived relations among modules, but such relations should not be interpreted as causal effects or validated intervention targets in cross-sectional data. Future studies should combine repeated psychometric assessment with standardized multi-night PSG and use longitudinal or intensive time-series models, causal graphical analyses based on explicitly stated temporal and confounding assumptions, and, where feasible, experimental or intervention designs. Sixth, the psychometric data partially overlap with a separate insomnia-focused sample reported elsewhere, although the PSG data, node configuration, and analytical framework are entirely distinct, and this overlap has been disclosed. Finally, the HAMD weight and diurnal variation factors showed floor effects; the expected influence ranking was largely unchanged after their removal, but estimates for these two nodes should still be interpreted with caution.

## 5 Conclusion

This study integrated psychometric and PSG indicators in a multimodal Gaussian graphical model and identified stronger within-modality associations alongside a limited number of small cross-modal conditional associations. Psychic anxiety and log(AHI + 1) showed the largest-magnitude bridge expected influence values within their respective modalities, followed by medication beliefs, but these connectivity statistics do not identify causal mechanisms or treatment targets. The findings suggest that psychometric assessment and PSG may provide complementary rather than interchangeable information in sleep-center patients. Given the retrospective, nonpreregistered design and the absence of prospective sample-size planning, these findings should be regarded as exploratory and hypothesis-generating. Longitudinal studies incorporating closer temporal alignment, repeated PSG measurements, and intervention designs are needed to determine the origin and clinical significance of these cross-modal patterns.

## Data Availability

The raw data supporting the conclusions of this article will be made available by the authors, without undue reservation.

## References

[1] Benjafield AV Ayas NT Eastwood PR Heinzer R Ip MSM Morrell MJ . Estimation of the global prevalence and burden of obstructive sleep apnoea: a literature-based analysis. Lancet Respir Med. (2019) 7:687–98. doi: 10.1016/S2213-2600(19)30198-5 31300334 PMC7007763

[2] Benjafield AV Sert Kuniyoshi FH Malhotra A Martin JL Morin CM Maurer LF . Estimation of the global prevalence and burden of insomnia: a systematic literature review-based analysis. Sleep Med Rev. (2025) 82:102121. doi: 10.1016/j.smrv.2025.102121 40627924 PMC12676268

[3] Sweetman A Lack L Bastien C . Co-morbid insomnia and sleep apnea (COMISA): prevalence, consequences, methodological considerations, and recent randomized controlled trials. Brain Sci. (2019) 9:371. doi: 10.3390/brainsci9120371 31842520 PMC6956217

[4] Garbarino S Bardwell WA Guglielmi O Chiorri C Bonanni E Magnavita N . Association of anxiety and depression in obstructive sleep apnea patients: a systematic review and meta-analysis. Behav Sleep Med. (2020) 18:35–57. doi: 10.1080/15402002.2018.1545649 30453780

[5] Baglioni C Battagliese G Feige B Spiegelhalder K Nissen C Voderholzer U . Insomnia as a predictor of depression: a meta-analytic evaluation of longitudinal epidemiological studies. J Affect Disord. (2011) 135:10–9. doi: 10.1016/j.jad.2011.01.011 21300408

[6] Harvey AG . A cognitive model of insomnia. Behav Res Ther. (2002) 40:869–93. doi: 10.1016/S0005-7967(01)00061-4 12186352

[7] Harvey AG Tang NKY . (Mis)perception of sleep in insomnia: a puzzle and a resolution. psychol Bull. (2012) 138:77–101. doi: 10.1037/a0025730 21967449 PMC3277880

[8] Baglioni C Regen W Teghen A Spiegelhalder K Feige B Nissen C . Sleep changes in the disorder of insomnia: a meta-analysis of polysomnographic studies. Sleep Med Rev. (2014) 18:195–213. doi: 10.1016/j.smrv.2013.04.001 23809904

[9] Rezaie L Fobian AD McCall WV Khazaie H . Paradoxical insomnia and subjective–objective sleep discrepancy: a review. Sleep Med Rev. (2018) 40:196–202. doi: 10.1016/j.smrv.2018.01.002 29402512

[10] Bjorvatn B Rajakulendren N Lehmann S Pallesen S . Increased severity of obstructive sleep apnea is associated with less anxiety and depression. J Sleep Res. (2018) 27:e12647. doi: 10.1111/jsr.12647 29193447

[11] Liu Y Zhu Z Yan J Wang Y . The relationship between anxiety and depression symptoms in insomnia patients: a network analysis. Sci Rep. (2025) 15:25030. doi: 10.1038/s41598-025-09746-w 40646108 PMC12254401

[12] Xing H Ma X Meng F Li Z . Fatigue as a moderator in symptom networks of insomnia, anxiety, and depression: insights from moderated network analysis. Front Psychiatry. (2025) 16:1644015. doi: 10.3389/fpsyt.2025.1644015 41532059 PMC12794031

[13] Luo X Li S Wu Q Xu Y Fang R Cheng Y . Depressive, anxiety, and sleep disturbance symptoms in patients with obstructive sleep apnea: a network analysis perspective. BMC Psychiatry. (2025) 25:77. doi: 10.1186/s12888-025-06532-w 39875912 PMC11773896

[14] Morin CM Vallières A Ivers H . Dysfunctional beliefs and attitudes about sleep (DBAS): validation of a brief version (DBAS-16). Sleep. (2007) 30:1547–54. doi: 10.1093/sleep/30.11.1547 18041487 PMC2082102

[15] Hertenstein E Nissen C Riemann D Feige B Baglioni C Spiegelhalder K . The exploratory power of sleep effort, dysfunctional beliefs and arousal for insomnia severity and polysomnography‐determined sleep. J Sleep Res. (2015) 24:399–406. doi: 10.1111/jsr.12293 25773981

[16] Fried EI Nesse RM . Depression sum-scores don’t add up: why analyzing specific depression symptoms is essential. BMC Med. (2015) 13:72. doi: 10.1186/s12916-015-0325-4 25879936 PMC4386095

[17] Chung K-F Ho F-Y Yeung W-F . Psychometric comparison of the full and abbreviated versions of the dysfunctional beliefs and attitudes about sleep scale. J Clin Sleep Med. (2016) 12:821–8. doi: 10.5664/jcsm.5878 26857054 PMC4877314

[18] Borsboom D Deserno MK Rhemtulla M Epskamp S Fried EI McNally RJ . Network analysis of multivariate data in psychological science. Nat Rev Methods Primers. (2021) 1:58. doi: 10.1038/s43586-021-00055-w 37880705

[19] Isvoranu A-M Abdin E Chong SA Vaingankar J Borsboom D Subramaniam M . Extended network analysis: from psychopathology to chronic illness. BMC Psychiatry. (2021) 21:119. doi: 10.1186/s12888-021-03128-y 33639891 PMC7913444

[20] Hamilton M . The assessment of anxiety states by rating. Br J Med Psychol. (1959) 32:50–5. doi: 10.1111/j.2044-8341.1959.tb00467.x 13638508

[21] Hamilton M . A rating scale for depression. J Neurology Neurosurg Psychiatry. (1960) 23:56–62. doi: 10.1136/jnnp.23.1.56 14399272 PMC495331

[22] Berry R Quan S Abreu A . The AASM Manual for the Scoring of Sleep and Associated Events: Rules, Terminology and Technical Specifications, Version 2.6. Darien, IL: American Academy of Sleep Medicine (2020).

[23] R Core Team . R: A Language and Environment for Statistical Computing. Vienna, Austria: R Foundation for Statistical Computing (2025). Available online at: https://www.R-project.org/ (Accessed August 25, 2026).

[24] Scrucca L Fop M Murphy TB Raftery AE . mclust 5: clustering, classification and density estimation using gaussian finite mixture models. R J. (2016) 8:289. doi: 10.32614/RJ-2016-021 27818791 PMC5096736

[25] Jones PJ Ma R McNally RJ . Bridge centrality: a network approach to understanding comorbidity. Multivar Behav Res. (2021) 56:353–67. doi: 10.1080/00273171.2019.1614898 31179765

[26] Epskamp S Borsboom D Fried EI . Estimating psychological networks and their accuracy: a tutorial paper. Behav Res. (2018) 50:195–212. doi: 10.3758/s13428-017-0862-1 28342071 PMC5809547

[27] Robinaugh DJ Millner AJ McNally RJ . Identifying highly influential nodes in the complicated grief network. J Abnormal Psychol. (2016) 125:747–57. doi: 10.1037/abn0000181 27505622 PMC5060093

[28] Epskamp S Cramer AOJ Waldorp LJ Schmittmann VD Borsboom D . qgraph: network visualizations of relationships in psychometric data. J Stat Soft. (2012) 48(4):1–18. doi: 10.18637/jss.v048.i04

[29] Van Borkulo CD Van Bork R Boschloo L Kossakowski JJ Tio P Schoevers RA . Comparing network structures on three aspects: a permutation test. Psychol Methods. (2023) 28:1273–85. doi: 10.1037/met0000476 35404628

[30] Haslbeck JMB Waldorp LJ . mgm: estimating time-varying mixed graphical models in high-dimensional data. J Stat Soft. (2020) 93(8):1–46. doi: 10.18637/jss.v093.i08

[31] Hu S Shi L Li Z Ma Y Li J Bao Y . First‐night effect in insomnia disorder: a systematic review and meta‐analysis of polysomnographic findings. J Sleep Res. (2024) 33:e13942. doi: 10.1111/jsr.13942 37254247

[32] Newell J Mairesse O Verbanck P Neu D . Is a one-night stay in the lab really enough to conclude? First-night effect and night-to-night variability in polysomnographic recordings among different clinical population samples. Psychiatry Res. (2012) 200:795–801. doi: 10.1016/j.psychres.2012.07.045 22901399

[33] De Mendonça FMR De Mendonça GPRR Souza LC Galvão LP Paiva HS De Azevedo Marques Périco C . Benzodiazepines and sleep architecture: a systematic review. CNSNDDT. (2023) 22:172–9. doi: 10.2174/1871527320666210618103344 34145997

[34] Song Y Kelly MR Fung CH Dzierzewski JM Grinberg AM Mitchell MN . Change in dysfunctional sleep-related beliefs is associated with changes in sleep and other health outcomes among older veterans with insomnia: findings from a randomized controlled trial. Ann Behav Med. (2022) 56:35–49. doi: 10.1093/abm/kaab030 33944909 PMC8691391

[35] Trimmel K Eder HG Böck M Stefanic-Kejik A Klösch G Seidel S . The (mis)perception of sleep: factors influencing the discrepancy between self-reported and objective sleep parameters. J Clin Sleep Med. (2021) 17:917–24. doi: 10.5664/jcsm.9086 33393901 PMC8320481

[36] Tein J-Y Coxe S Cham H . Statistical power to detect the correct number of classes in latent profile analysis. Struct Equation Modeling: A Multidiscip J. (2013) 20:640–57. doi: 10.1080/10705511.2013.824781 24489457 PMC3904803

[37] Ekwaru JP Veugelers PJ . The overlooked importance of constants added in log transformation of independent variables with zero values: a proposed approach for determining an optimal constant. Stat Biopharm Res. (2018) 10:26–9. doi: 10.1080/19466315.2017.1369900 37339054

[38] Pan C Zhang Q Zhu Y Kong S Liu J Zhang C . Module control of network analysis in psychopathology. iScience. (2024) 27:110302. doi: 10.1016/j.isci.2024.110302 39045106 PMC11263636

