# Supplementary material for: Cross-modal conditional associations between psychometric and polysomnographic indicators in sleep center patients: a Gaussian graphical model
Source: Front Psychiatry. 2026 Sep 3;17:1907602. doi: 10.3389/fpsyt.2026.1907602 (PMC13582383; doi:10.3389/fpsyt.2026.1907602)
Supplement: Supplementary file 1 [file Supplementaryfile1.docx]

**Supplementary Material**

**Table S1A.** Data dictionary for all variables used in the analyses.

| **Variable** | **Role** | **Definition or source** | **Direction of higher values** |
| --- | --- | --- | --- |
| HA_Psy | Factor score | sum(raw items 1,2,3,4,5,6,14) | higher = more severe/more dysfunctional |
| HA_Som | Factor score | sum(raw items 7,8,9,10,11,12,13) | higher = more severe/more dysfunctional |
| HD_F1 | Factor score | sum(raw items 10,11,12,13,15,17) | higher = more severe/more dysfunctional |
| HD_F2 | Factor score | sum(raw items 16) | higher = more severe/more dysfunctional |
| HD_F3 | Factor score | sum(raw items 2,3,9,19,20,21) | higher = more severe/more dysfunctional |
| HD_F4 | Factor score | sum(raw items 18) | higher = more severe/more dysfunctional |
| HD_F5 | Factor score | sum(raw items 1,7,8,14) | higher = more severe/more dysfunctional |
| HD_F6 | Factor score | sum(raw items 4,5,6) | higher = more severe/more dysfunctional |
| HD_F7 | Factor score | sum(raw items 22,23,24) | higher = more severe/more dysfunctional |
| DB_F1 | Factor score | mean(6 - raw items 5,7,9,12,16) | higher = more severe/more dysfunctional |
| DB_F2 | Factor score | mean(6 - raw items 3,4,8,10,11,14) | higher = more severe/more dysfunctional |
| DB_F3 | Factor score | mean(6 - raw items 1,2) | higher = more severe/more dysfunctional |
| DB_F4 | Factor score | mean(6 - raw items 6,13,15) | higher = more severe/more dysfunctional |
| HAMA_total | scale total | sum HAMA items 1-14 | higher = more anxiety |
| HAMD24_total | scale total | sum HAMD items 1-24 | higher = more depression |
| DBAS16_raw_sum | scale total | sum DBAS raw items 1-16 | lower = more dysfunctional |
| DBAS16_reversed_sum | scale total | sum(6 - DBAS raw items) | higher = more dysfunctional |
| DBAS16_reversed_mean | scale total | mean(6 - DBAS raw items) | higher = more dysfunctional |
| SE | PSG/derived | frozen main/PSG merge SE | as measured |
| AHI_raw | PSG/derived | frozen main/PSG merge AHI | as measured |
| AHI_log | PSG/derived | log1p(AHI_raw) | as measured |
| AHI_log_z | PSG/derived | sample z-score of AHI_log | as measured |
| ArI | PSG/derived | frozen main/PSG merge ArI | as measured |
| N3pct | PSG/derived | frozen main/PSG merge N3 | as measured |
| REMpct | PSG/derived | frozen main/PSG merge REM | as measured |
| TST | PSG/derived | frozen main/PSG merge TST | as measured |

Direction indicates the clinical meaning of higher values. All DBAS-16 items were reverse scored before analysis, so higher values on every psychometric indicator denote greater symptom severity or more dysfunctional sleep beliefs.

**Table S1B.** Item-to-factor mapping for the HAMA-14, HAMD-24, and DBAS-16.

| **Factor** | **Scale** | **Label** | **Items** | **Scoring** | **Direction** | **Theoretical range** |
| --- | --- | --- | --- | --- | --- | --- |
| HA_Psy | HAMA | Psychic anxiety | 1,2,3,4,5,6,14 | item sum | higher = more severe/more dysfunctional | 0-28 |
| HA_Som | HAMA | Somatic anxiety | 7,8,9,10,11,12,13 | item sum | higher = more severe/more dysfunctional | 0-28 |
| HD_F1 | HAMD | Anxiety/somatization | 10,11,12,13,15,17 | item sum | higher = more severe/more dysfunctional | 0-18 |
| HD_F2 | HAMD | Weight | 16 | item sum | higher = more severe/more dysfunctional | 0-2 |
| HD_F3 | HAMD | Cognitive impairment | 2,3,9,19,20,21 | item sum | higher = more severe/more dysfunctional | 0-22 |
| HD_F4 | HAMD | Diurnal variation | 18 | item sum | higher = more severe/more dysfunctional | 0-2 |
| HD_F5 | HAMD | Retardation | 1,7,8,14 | item sum | higher = more severe/more dysfunctional | 0-14 |
| HD_F6 | HAMD | Sleep disturbance | 4,5,6 | item sum | higher = more severe/more dysfunctional | 0-6 |
| HD_F7 | HAMD | Hopelessness | 22,23,24 | item sum | higher = more severe/more dysfunctional | 0-12 |
| DB_F1 | DBAS | Consequences of insomnia | 5,7,9,12,16 | mean(6 - original item score) | higher = more severe/more dysfunctional | 1-5 |
| DB_F2 | DBAS | Worry/helplessness about sleep | 3,4,8,10,11,14 | mean(6 - original item score) | higher = more severe/more dysfunctional | 1-5 |
| DB_F3 | DBAS | Sleep expectations | 1,2 | mean(6 - original item score) | higher = more severe/more dysfunctional | 1-5 |
| DB_F4 | DBAS | Medication beliefs | 6,13,15 | mean(6 - original item score) | higher = more severe/more dysfunctional | 1-5 |

Item numbers refer to the position of each item within its own questionnaire. Direction refers to the meaning of higher factor scores after reverse scoring of the DBAS-16.

**Table S2.** Internal consistency, theoretical and observed ranges, and 95% confidence intervals.

| **Code** | **Factor** | **Scale** | **Items** | **Method** | **Theoretical range** | **Observed range** | **Estimate** | **95% CI** |
| --- | --- | --- | --- | --- | --- | --- | --- | --- |
| HAMA14 | HAMA-14 total | HAMA | 14 | Ordinal omega | 0-56 | 2.00 to 49.00 | 0.883 | 0.855 to 0.907 |
| HA_Psy | Psychic anxiety | HAMA | 7 | Ordinal omega | 0-28 | 0.00 to 24.00 | 0.821 | 0.784 to 0.854 |
| HA_Som | Somatic anxiety | HAMA | 7 | Ordinal omega | 0-28 | 0.00 to 25.00 | 0.802 | 0.746 to 0.845 |
| HAMD24 | HAMD-24 total | HAMD | 24 | Ordinal omega | 0-76 | 0.00 to 48.00 | 0.879 | 0.853 to 0.901 |
| HD_F1 | Anxiety/somatization | HAMD | 6 | Ordinal omega | 0-18 | 0.00 to 14.00 | 0.595 | 0.501 to 0.673 |
| HD_F2 | Weight | HAMD | 1 | Not applicable (single item) | 0-2 | 0.00 to 2.00 | — | — |
| HD_F3 | Cognitive impairment | HAMD | 6 | Ordinal omega | 0-22 | 0.00 to 14.00 | 0.731 | 0.652 to 0.796 |
| HD_F4 | Diurnal variation | HAMD | 1 | Not applicable (single item) | 0-2 | 0.00 to 2.00 | — | — |
| HD_F5 | Retardation | HAMD | 4 | Ordinal omega | 0-14 | 0.00 to 9.00 | 0.590 | 0.496 to 0.686 |
| HD_F6 | Sleep disturbance | HAMD | 3 | Ordinal omega | 0-6 | 0.00 to 6.00 | 0.663 | 0.571 to 0.748 |
| HD_F7 | Hopelessness | HAMD | 3 | Ordinal omega | 0-12 | 0.00 to 8.00 | 0.721 | 0.661 to 0.786 |
| DBAS16 | DBAS-16 reversed mean | DBAS | 16 | Ordinal omega | 1-5 | 1.38 to 4.88 | 0.896 | 0.870 to 0.917 |
| DB_F1 | Consequences of insomnia | DBAS | 5 | Ordinal omega | 1-5 | 1.00 to 5.00 | 0.832 | 0.788 to 0.871 |
| DB_F2 | Worry/helplessness about sleep | DBAS | 6 | Ordinal omega | 1-5 | 1.17 to 5.00 | 0.819 | 0.767 to 0.862 |
| DB_F3 | Sleep expectations | DBAS | 2 | Spearman-Brown (polychoric) | 1-5 | 1.00 to 5.00 | 0.513 | 0.348 to 0.640 |
| DB_F4 | Medication beliefs | DBAS | 3 | Ordinal omega | 1-5 | 1.33 to 5.00 | 0.653 | 0.575 to 0.729 |

Ordinal McDonald's omega was used for factors with three or more items and the Spearman-Brown coefficient for two-item factors. Confidence intervals are percentile intervals from 1,000 bootstrap resamples, all of which completed successfully. Single-item factors have no internal consistency estimate.

**Table S3.** Descriptive statistics for the 17 final network variables and the untransformed AHI.

| **Variable** | **Modality** | **Mean (SD)** | **Median** | **IQR** | **Range** | **Zero values, n (%)** | **Unique values** | **Skewness** | **Excess kurtosis** |
| --- | --- | --- | --- | --- | --- | --- | --- | --- | --- |
| HA_Psy | Psychometric | 9.12 (4.30) | 9.00 | 6.00 | 0.00 to 24.00 | 1 (0.3) | 23 | 0.67 | 0.53 |
| HA_Som | Psychometric | 6.10 (3.78) | 5.00 | 4.00 | 0.00 to 25.00 | 10 (3.1) | 21 | 1.20 | 2.88 |
| HD_F2 | Psychometric | 0.36 (0.66) | 0.00 | 1.00 | 0.00 to 2.00 | 235 (73.9) | 3 | 1.57 | 1.07 |
| HD_F3 | Psychometric | 2.28 (2.08) | 2.00 | 2.00 | 0.00 to 14.00 | 64 (20.1) | 11 | 1.49 | 3.95 |
| HD_F4 | Psychometric | 0.46 (0.60) | 0.00 | 1.00 | 0.00 to 2.00 | 190 (59.7) | 3 | 0.92 | -0.16 |
| HD_F5 | Psychometric | 2.17 (1.76) | 2.00 | 2.00 | 0.00 to 9.00 | 66 (20.8) | 10 | 0.76 | 0.48 |
| HD_F6 | Psychometric | 3.82 (1.45) | 4.00 | 2.00 | 0.00 to 6.00 | 9 (2.8) | 7 | -0.65 | -0.23 |
| HD_F7 | Psychometric | 2.08 (1.85) | 2.00 | 2.00 | 0.00 to 8.00 | 75 (23.6) | 9 | 0.79 | -0.02 |
| DB_F1 | Psychometric | 3.42 (0.66) | 3.60 | 1.00 | 1.00 to 5.00 | 0 (0.0) | 18 | -0.53 | 0.19 |
| DB_F2 | Psychometric | 3.56 (0.54) | 3.67 | 0.67 | 1.17 to 5.00 | 0 (0.0) | 18 | -0.69 | 1.52 |
| DB_F3 | Psychometric | 3.24 (0.80) | 3.50 | 1.50 | 1.00 to 5.00 | 0 (0.0) | 9 | -0.17 | -0.53 |
| DB_F4 | Psychometric | 3.01 (0.66) | 3.00 | 0.67 | 1.33 to 5.00 | 0 (0.0) | 12 | 0.07 | -0.50 |
| SE | PSG | 68.30 (15.65) | 70.50 | 23.07 | 22.50 to 96.00 | 0 (0.0) | 236 | -0.60 | -0.17 |
| AHI_log | PSG | 2.39 (1.03) | 2.46 | 1.39 | 0.00 to 4.46 | 2 (0.6) | 221 | -0.28 | -0.59 |
| ArI | PSG | 25.83 (14.20) | 22.40 | 16.38 | 2.60 to 91.90 | 0 (0.0) | 223 | 1.59 | 3.51 |
| N3pct | PSG | 10.18 (9.54) | 8.60 | 14.72 | 0.00 to 60.20 | 55 (17.3) | 175 | 1.08 | 1.73 |
| REMpct | PSG | 16.98 (7.76) | 17.40 | 9.60 | 0.00 to 42.70 | 8 (2.5) | 186 | 0.02 | 0.08 |
| AHI_raw | PSG_raw | 16.28 (16.61) | 10.75 | 17.20 | 0.00 to 85.60 | 2 (0.6) | 221 | 1.74 | 3.20 |

No values were missing for any variable (N = 318). Zero values are counts of exact zeros on the original measurement scale. The untransformed AHI is shown for reference only; the network used log(AHI + 1) as the node.

**Table S4A.** Matching intervals and temporal-window subsamples.

| **Window** | **Maximum gap, days** | **N** | **% of main sample** | **Network estimated** | **Definition** |
| --- | --- | --- | --- | --- | --- |
| Within 7 days (main sample) | 7 | 318 | 100.0 | Yes | All matched records within 7 days |
| Within 3 days | 3 | 312 | 98.1 | Yes | All matched records within 3 days |
| Within 1 day | 1 | 258 | 81.1 | Yes | All matched records within 1 day |
| Same day (descriptive only) | 0 | 116 | 36.5 | No | Same-day records; descriptive only |

The interval is the largest absolute number of days between the PSG date and the dates of the three questionnaires.

**Table S4B.** Distribution of the maximum psychometric-to-PSG interval.

| **Maximum absolute gap, days** | **n** | **%** |
| --- | --- | --- |
| 0 | 116 | 36.5 |
| 1 | 142 | 44.7 |
| 2 | 38 | 11.9 |
| 3 | 16 | 5.0 |
| 4 | 2 | 0.6 |
| 5 | 3 | 0.9 |
| 7 | 1 | 0.3 |

Percentages are of the full analytic sample (N = 318).

**Table S5A.** Latent profile analysis fit diagnostics for one- to six-class solutions.

| **Classes** | **npar** | **logLik** | **AIC** | **BIC** | **aBIC** | **Entropy** | **Smallest class, n (%)** | **Min. average posterior** | **Eligibility** |
| --- | --- | --- | --- | --- | --- | --- | --- | --- | --- |
| 1 | 36 | -8,112.99 | 16,297.98 | 16,433.41 | 16,319.23 | 1.000 | 318 (100.0) | 1.000 | Eligible |
| 2 | 56 | -7,709.63 | 15,531.25 | 15,741.93 | 15,564.31 | 0.862 | 124 (39.0) | 0.946 | Eligible |
| 3 | 76 | -7,533.40 | 15,218.80 | 15,504.72 | 15,263.66 | 0.878 | 54 (17.0) | 0.936 | Eligible |
| 4 | 96 | -7,423.51 | 15,039.03 | 15,400.19 | 15,095.69 | 0.898 | 28 (8.8) | 0.915 | Eligible |
| 5 | 116 | -7,328.63 | 14,889.26 | 15,325.66 | 14,957.73 | 0.897 | 20 (6.3) | 0.913 | Eligible |
| 6 | 136 | -7,279.17 | 14,830.33 | 15,341.97 | 14,910.61 | 0.894 | 13 (4.1) | 0.906 | Smallest class below 5% |

All models shown used the selected varying-volume, equal-shape, diagonal (VEI) covariance structure and all converged without singular covariance matrices, empty classes, or numerical anomalies. For the one-class model the algorithm returned the equivalent XXI parameterization. Selection required entropy of at least 0.80 and a smallest class of at least 5% of the sample, applied together, and was restricted to the six parameterizations with diagonal covariance matrices. The comparison of all 14 covariance parameterizations is reported in Table S10. npar, number of estimated parameters.

**Table S5B.** Classification diagnostics for the selected five-class solution.

| **Class** | **n** | **Proportion** | **Average posterior probability** |
| --- | --- | --- | --- |
| 1 | 40 | 0.126 | 0.970 |
| 2 | 115 | 0.362 | 0.940 |
| 3 | 26 | 0.082 | 0.936 |
| 4 | 20 | 0.063 | 0.980 |
| 5 | 117 | 0.368 | 0.913 |

Average posterior probability is the mean posterior probability of assignment among patients assigned to each class.

**Table S6A.** Eight-indicator sensitivity latent profile analysis fit diagnostics.

| **Classes** | **npar** | **logLik** | **AIC** | **BIC** | **aBIC** | **Entropy** | **Smallest class, n (%)** | **Eligibility** |
| --- | --- | --- | --- | --- | --- | --- | --- | --- |
| 1 | 16 | -3,605.77 | 7,243.55 | 7,303.74 | 7,252.99 | 1.000 | 318 (100.0) | Eligible |
| 2 | 32 | -3,453.08 | 6,970.16 | 7,090.55 | 6,989.05 | 0.818 | 75 (23.6) | Eligible |
| 3 | 48 | -3,368.80 | 6,833.60 | 7,014.18 | 6,861.93 | 0.748 | 56 (17.6) | Entropy below 0.80 |
| 4 | 64 | -3,310.98 | 6,749.96 | 6,990.74 | 6,787.74 | 0.782 | 30 (9.4) | Entropy below 0.80 |
| 5 | 80 | -3,211.84 | 6,583.68 | 6,884.65 | 6,630.90 | 0.828 | 35 (11.0) | Eligible |
| 6 | 96 | -3,220.63 | 6,633.26 | 6,994.42 | 6,689.92 | 0.797 | 22 (6.9) | Entropy below 0.80 |

The 13 psychometric indicators were replaced by the HAMA-14 total, the HAMD-24 total, and the reverse-scored DBAS-16 mean, combined with the 5 PSG indicators. The same covariance parameterization comparison and selection rules were applied as in the main analysis, and the varying-volume, varying-shape, diagonal (EVI) specification was selected; all models shown used that parameterization.

**Table S6B.** Agreement between the main and eight-indicator classifications.

| **Metric** | **Value** |
| --- | --- |
| Patients compared | 318 |
| Classes in the main solution | 5 |
| Classes in the eight-indicator solution | 5 |
| Adjusted Rand index | 0.264 |

The adjusted Rand index compares the two classifications of the same 318 patients.

**Table S6C.** Cross-tabulation of the main and eight-indicator classifications.

| **Main solution class** | **Eight-indicator class** | **n** |
| --- | --- | --- |
| 1 | 1 | 0 |
| 2 | 1 | 21 |
| 3 | 1 | 17 |
| 4 | 1 | 0 |
| 5 | 1 | 3 |
| 1 | 2 | 2 |
| 2 | 2 | 29 |
| 3 | 2 | 6 |
| 4 | 2 | 2 |
| 5 | 2 | 23 |
| 1 | 3 | 7 |
| 2 | 3 | 2 |
| 3 | 3 | 0 |
| 4 | 3 | 18 |
| 5 | 3 | 8 |
| 1 | 4 | 0 |
| 2 | 4 | 60 |
| 3 | 4 | 3 |
| 4 | 4 | 0 |
| 5 | 4 | 14 |
| 1 | 5 | 31 |
| 2 | 5 | 3 |
| 3 | 5 | 0 |
| 4 | 5 | 0 |
| 5 | 5 | 69 |

Classes of the eight-indicator solution are numbered independently of the main solution and do not correspond to C1 through C5.

**Table S7.** Goldbricker redundancy screening of all 78 psychometric node pairs.

| **Node 1** | **Node 2** | **Zero-order r** | **Proportion significantly different** | **Candidate** | **Action** |
| --- | --- | --- | --- | --- | --- |
| DB_F1 | DB_F2 | 0.716 | 0.062 | Yes | Retain both nodes; require later node-set sensitivity interpretation |
| HA_Som | HD_F1 | 0.611 | 0.062 | Yes | Resolved by removing HD_F1 from the final network node set |
| HD_F3 | HD_F5 | 0.606 | 0.000 | Yes | Retain both nodes; require later node-set sensitivity interpretation |
| HA_Psy | HD_F1 | 0.597 | 0.125 | Yes | Resolved by removing HD_F1 from the final network node set |
| HD_F5 | HD_F7 | 0.583 | 0.000 | Yes | Retain both nodes; require later node-set sensitivity interpretation |
| HD_F3 | HD_F7 | 0.573 | 0.000 | Yes | Retain both nodes; require later node-set sensitivity interpretation |
| HD_F1 | HD_F7 | 0.527 | 0.062 | Yes | Resolved by removing HD_F1 from the final network node set |
| HD_F1 | HD_F5 | 0.521 | 0.188 | Yes | Resolved by removing HD_F1 from the final network node set |
| HD_F1 | HD_F3 | 0.514 | 0.062 | Yes | Resolved by removing HD_F1 from the final network node set |
| HA_Psy | HA_Som | 0.673 | 0.312 | No | — |
| HA_Psy | HD_F5 | 0.604 | 0.250 | No | — |
| HA_Psy | HD_F3 | 0.564 | 0.500 | No | — |
| HA_Psy | HD_F7 | 0.537 | 0.250 | No | — |
| DB_F2 | DB_F4 | 0.525 | 0.250 | No | — |
| DB_F1 | DB_F4 | 0.492 | 0.312 | No | — |
| HA_Som | HD_F7 | 0.489 | 0.250 | No | — |
| HA_Som | HD_F5 | 0.484 | 0.250 | No | — |
| HA_Som | HD_F3 | 0.456 | 0.312 | No | — |
| HD_F4 | HD_F7 | 0.364 | 0.312 | No | — |
| HA_Psy | HD_F4 | 0.361 | 0.500 | No | — |
| DB_F1 | DB_F3 | 0.326 | 0.438 | No | — |
| HD_F3 | HD_F4 | 0.323 | 0.312 | No | — |
| HD_F1 | HD_F4 | 0.320 | 0.312 | No | — |
| HA_Psy | DB_F2 | 0.318 | 0.688 | No | — |
| DB_F2 | DB_F3 | 0.318 | 0.500 | No | — |
| HA_Psy | DB_F1 | 0.307 | 0.688 | No | — |
| HD_F6 | DB_F2 | 0.303 | 0.188 | No | — |
| HD_F4 | HD_F5 | 0.299 | 0.312 | No | — |
| HA_Som | HD_F4 | 0.296 | 0.312 | No | — |
| HA_Psy | HD_F6 | 0.294 | 0.500 | No | — |
| DB_F3 | DB_F4 | 0.290 | 0.375 | No | — |
| HD_F3 | DB_F1 | 0.269 | 0.500 | No | — |
| HD_F6 | DB_F1 | 0.268 | 0.188 | No | — |
| HD_F1 | DB_F2 | 0.237 | 0.562 | No | — |
| HD_F3 | DB_F3 | 0.234 | 0.562 | No | — |
| HD_F5 | DB_F1 | 0.230 | 0.625 | No | — |
| HD_F1 | DB_F1 | 0.219 | 0.625 | No | — |
| HD_F2 | DB_F2 | 0.214 | 0.438 | No | — |
| HD_F3 | DB_F2 | 0.213 | 0.500 | No | — |
| HD_F7 | DB_F1 | 0.210 | 0.688 | No | — |
| HD_F4 | HD_F6 | 0.209 | 0.375 | No | — |
| HD_F6 | DB_F4 | 0.207 | 0.250 | No | — |
| HA_Som | DB_F1 | 0.206 | 0.625 | No | — |
| HD_F2 | DB_F1 | 0.203 | 0.312 | No | — |
| HA_Som | DB_F2 | 0.201 | 0.625 | No | — |
| HD_F3 | DB_F4 | 0.199 | 0.500 | No | — |
| HD_F2 | DB_F4 | 0.192 | 0.312 | No | — |
| HD_F4 | DB_F2 | 0.189 | 0.375 | No | — |
| HD_F2 | HD_F7 | 0.186 | 0.438 | No | — |
| HA_Psy | HD_F2 | 0.183 | 0.438 | No | — |
| HD_F1 | HD_F2 | 0.181 | 0.438 | No | — |
| HD_F5 | DB_F3 | 0.180 | 0.562 | No | — |
| HD_F1 | HD_F6 | 0.176 | 0.375 | No | — |
| HD_F4 | DB_F1 | 0.175 | 0.312 | No | — |
| HD_F3 | HD_F6 | 0.172 | 0.375 | No | — |
| HD_F1 | DB_F3 | 0.167 | 0.688 | No | — |
| HD_F5 | DB_F2 | 0.165 | 0.562 | No | — |
| HA_Som | HD_F2 | 0.158 | 0.500 | No | — |
| HD_F4 | DB_F3 | 0.158 | 0.500 | No | — |
| HD_F7 | DB_F2 | 0.149 | 0.688 | No | — |
| HD_F7 | DB_F3 | 0.142 | 0.625 | No | — |
| HA_Som | HD_F6 | 0.140 | 0.438 | No | — |
| HD_F2 | HD_F3 | 0.135 | 0.438 | No | — |
| HD_F4 | DB_F4 | 0.132 | 0.500 | No | — |
| HD_F2 | HD_F5 | 0.127 | 0.438 | No | — |
| HD_F5 | HD_F6 | 0.125 | 0.375 | No | — |
| HD_F7 | DB_F4 | 0.118 | 0.625 | No | — |
| HD_F2 | HD_F4 | 0.117 | 0.312 | No | — |
| HA_Som | DB_F3 | 0.115 | 0.500 | No | — |
| HD_F2 | HD_F6 | 0.112 | 0.000 | No | — |
| HA_Psy | DB_F3 | 0.110 | 0.688 | No | — |
| HD_F5 | DB_F4 | 0.107 | 0.500 | No | — |
| HD_F1 | DB_F4 | 0.102 | 0.562 | No | — |
| HD_F6 | HD_F7 | 0.101 | 0.438 | No | — |
| HA_Psy | DB_F4 | 0.074 | 0.688 | No | — |
| HA_Som | DB_F4 | 0.036 | 0.625 | No | — |
| HD_F2 | DB_F3 | -0.028 | 0.062 | No | — |
| HD_F6 | DB_F3 | 0.015 | 0.188 | No | — |

Rows are ordered by candidate status and then by the absolute zero-order correlation. A pair met the candidate criterion when the proportion of significantly different correlations fell below 0.25 and the zero-order correlation reached at least 0.50. Content adjudication was applied only to candidate pairs; all other pairs retained both nodes without adjudication.

**Table S8A.** Network summary for the main and sensitivity specifications.

| **Network** | **N** | **Nodes** | **Possible edges** | **Non-zero edges** | **Density** | **Positive / negative** | **Cross-modal non-zero / possible** |
| --- | --- | --- | --- | --- | --- | --- | --- |
| Main network (17 nodes, gamma = 0.50) | 318 | 17 | 136 | 43 | 0.316 | 37 / 6 | 3 / 60 |
| Floor-effect sensitivity (16 nodes) | 318 | 16 | 120 | 44 | 0.367 | 33 / 11 | 8 / 55 |
| Regularization sensitivity (gamma = 0) | 318 | 17 | 136 | 55 | 0.404 | 41 / 14 | 11 / 60 |
| Spearman sensitivity (raw AHI ranks) | 318 | 17 | 136 | 54 | 0.397 | 42 / 12 | 10 / 60 |

Density is the number of non-zero edges divided by the number of possible edges. The floor-effect network excludes the two single-item HAMD factors and returns the anxiety/somatization factor, so its node set and possible-edge count differ from the main network.

**Table S8B.** Comparison of the sensitivity networks with the main network.

| **Network** | **Common nodes** | **Retained / lost / gained** | **Edge r (Pearson)** | **Edge r (Spearman)** | **MAD** | **Jaccard** | **Sign agreement** | **Cross-modal main / sensitivity** | **EI rank r** | **bEI rank r psychometric** | **bEI rank r PSG** |
| --- | --- | --- | --- | --- | --- | --- | --- | --- | --- | --- | --- |
| Floor-effect (16 nodes) | 15 | 31 / 0 / 7 | 0.994 | 0.936 | 0.006 | 0.816 | 1.000 | 3 / 8 | 0.993 | 0.823 | 0.872 |
| Regularization (gamma = 0) | 17 | 43 / 0 / 12 | 0.995 | 0.927 | 0.006 | 0.782 | 1.000 | 3 / 11 | 1.000 | 0.780 | 0.667 |
| Spearman | 17 | 43 / 0 / 11 | 0.977 | 0.922 | 0.010 | 0.796 | 1.000 | 3 / 10 | 0.966 | 0.766 | 0.564 |

Comparisons are restricted to node pairs common to both networks. MAD, mean absolute difference across the full edge weight vector. Sign agreement is the proportion of shared non-zero edges carrying the same sign. Rank correlations are Spearman coefficients; bridge expected influence is ranked within each modality separately.

**Table S8C.** Network summary for the temporal-window subsamples.

| **Network** | **N** | **Nodes** | **Possible edges** | **Non-zero edges** | **Density** | **Positive / negative** | **Cross-modal non-zero / possible** |
| --- | --- | --- | --- | --- | --- | --- | --- |
| Main sample (<= 7 days) | 318 | 17 | 136 | 43 | 0.316 | 37 / 6 | 3 / 60 |
| Temporal sensitivity (<= 3 days) | 312 | 17 | 136 | 42 | 0.309 | 36 / 6 | 3 / 60 |
| Temporal sensitivity (<= 1 day) | 258 | 17 | 136 | 44 | 0.324 | 37 / 7 | 4 / 60 |

Each network was re-estimated from scratch within the stated window using the same specification as the main network.

**Table S8D.** Comparison of the temporal-window networks with the main network.

| **Network** | **Common nodes** | **Retained / lost / gained** | **Edge r (Pearson)** | **Edge r (Spearman)** | **MAD** | **Jaccard** | **Sign agreement** | **Cross-modal main / sensitivity** | **EI rank r** | **bEI rank r psychometric** | **bEI rank r PSG** |
| --- | --- | --- | --- | --- | --- | --- | --- | --- | --- | --- | --- |
| <= 3 days | 17 | 41 / 2 / 1 | 0.999 | 0.976 | 0.001 | 0.932 | 1.000 | 3 / 3 | 0.998 | 0.988 | 0.895 |
| <= 1 day | 17 | 41 / 2 / 3 | 0.991 | 0.958 | 0.005 | 0.891 | 1.000 | 3 / 4 | 0.995 | 0.841 | 0.579 |

Comparisons are restricted to node pairs common to both networks. MAD, mean absolute difference across the full edge weight vector. Sign agreement is the proportion of shared non-zero edges carrying the same sign. Rank correlations are Spearman coefficients; bridge expected influence is ranked within each modality separately.

**Table S9.** Nonparametric edge-weight bootstrap results for all 136 possible edges.

| **Node 1** | **Node 2** | **Edge weight** | **Bootstrap mean** | **Bootstrap SE** | **95% percentile interval** | **Non-zero in network** | **Interval excludes zero** |
| --- | --- | --- | --- | --- | --- | --- | --- |
| DB_F1 | DB_F2 | 0.483 | 0.491 | 0.040 | 0.416 to 0.569 | Yes | Yes |
| AHI_log | ArI | 0.471 | 0.488 | 0.037 | 0.413 to 0.554 | Yes | Yes |
| HA_Psy | HA_Som | 0.392 | 0.397 | 0.041 | 0.319 to 0.472 | Yes | Yes |
| HD_F3 | HD_F5 | 0.255 | 0.256 | 0.051 | 0.163 to 0.354 | Yes | Yes |
| DB_F2 | DB_F4 | 0.228 | 0.227 | 0.047 | 0.132 to 0.316 | Yes | Yes |
| HD_F5 | HD_F7 | 0.224 | 0.225 | 0.053 | 0.119 to 0.323 | Yes | Yes |
| HA_Psy | HD_F5 | 0.222 | 0.224 | 0.038 | 0.146 to 0.296 | Yes | Yes |
| HD_F3 | HD_F7 | 0.219 | 0.223 | 0.049 | 0.127 to 0.316 | Yes | Yes |
| DB_F1 | DB_F4 | 0.164 | 0.163 | 0.049 | 0.065 to 0.257 | Yes | Yes |
| HA_Psy | HD_F3 | 0.158 | 0.158 | 0.045 | 0.072 to 0.245 | Yes | Yes |
| ArI | N3pct | -0.144 | -0.160 | 0.037 | -0.231 to -0.085 | Yes | Yes |
| HA_Som | HD_F7 | 0.126 | 0.126 | 0.049 | 0.035 to 0.230 | Yes | Yes |
| SE | REMpct | 0.121 | 0.134 | 0.064 | 0.000 to 0.257 | Yes | No |
| HD_F4 | HD_F7 | 0.119 | 0.119 | 0.047 | 0.028 to 0.212 | Yes | Yes |
| ArI | REMpct | -0.118 | -0.135 | 0.053 | -0.229 to -0.023 | Yes | Yes |
| SE | ArI | -0.097 | -0.111 | 0.050 | -0.206 to -0.014 | Yes | Yes |
| HA_Psy | HD_F4 | 0.097 | 0.083 | 0.043 | 0.000 to 0.171 | Yes | Yes |
| HD_F6 | DB_F2 | 0.095 | 0.091 | 0.044 | 0.003 to 0.178 | Yes | Yes |
| HA_Psy | HD_F7 | 0.095 | 0.094 | 0.043 | 0.007 to 0.179 | Yes | Yes |
| HA_Psy | HD_F6 | 0.092 | 0.097 | 0.038 | 0.021 to 0.173 | Yes | Yes |
| DB_F1 | DB_F3 | 0.084 | 0.087 | 0.049 | 0.000 to 0.189 | Yes | No |
| DB_F3 | DB_F4 | 0.073 | 0.083 | 0.052 | 0.000 to 0.188 | Yes | No |
| DB_F2 | DB_F3 | 0.065 | 0.069 | 0.050 | 0.000 to 0.173 | Yes | No |
| HA_Som | HD_F5 | 0.063 | 0.063 | 0.039 | 0.000 to 0.144 | Yes | No |
| HA_Psy | DB_F2 | 0.063 | 0.058 | 0.033 | 0.000 to 0.122 | Yes | No |
| HD_F3 | DB_F3 | 0.057 | 0.056 | 0.041 | 0.000 to 0.141 | Yes | No |
| HD_F3 | HD_F4 | 0.052 | 0.050 | 0.043 | 0.000 to 0.144 | Yes | No |
| HA_Som | HD_F3 | 0.046 | 0.046 | 0.038 | 0.000 to 0.126 | Yes | No |
| HA_Psy | DB_F1 | 0.040 | 0.038 | 0.033 | 0.000 to 0.109 | Yes | No |
| HD_F3 | DB_F1 | 0.039 | 0.032 | 0.031 | 0.000 to 0.103 | Yes | No |
| HA_Psy | AHI_log | -0.038 | -0.046 | 0.034 | -0.124 to 0.000 | Yes | No |
| HD_F2 | DB_F2 | 0.037 | 0.036 | 0.031 | 0.000 to 0.105 | Yes | No |
| DB_F4 | REMpct | -0.034 | -0.040 | 0.037 | -0.124 to 0.000 | Yes | No |
| HD_F4 | HD_F6 | 0.034 | 0.046 | 0.039 | 0.000 to 0.127 | Yes | No |
| HD_F6 | DB_F1 | 0.031 | 0.039 | 0.036 | 0.000 to 0.120 | Yes | No |
| HD_F2 | HD_F7 | 0.027 | 0.033 | 0.035 | 0.000 to 0.116 | Yes | No |
| HD_F2 | DB_F4 | 0.021 | 0.035 | 0.038 | 0.000 to 0.125 | Yes | No |
| HD_F2 | DB_F1 | 0.017 | 0.029 | 0.032 | 0.000 to 0.105 | Yes | No |
| HA_Som | HD_F4 | 0.016 | 0.031 | 0.037 | 0.000 to 0.124 | Yes | No |
| HA_Psy | HD_F2 | 0.012 | 0.017 | 0.025 | 0.000 to 0.083 | Yes | No |
| HD_F6 | SE | -0.010 | -0.036 | 0.040 | -0.126 to 0.000 | Yes | No |
| HD_F4 | HD_F5 | 0.003 | 0.024 | 0.034 | 0.000 to 0.108 | Yes | No |
| HA_Som | HD_F2 | 0 | 0.013 | 0.023 | 0.000 to 0.080 | No | No |
| HD_F2 | HD_F3 | 0 | 0.004 | 0.013 | 0.000 to 0.046 | No | No |
| HD_F2 | HD_F4 | 0 | 0.009 | 0.022 | 0.000 to 0.073 | No | No |
| HD_F2 | HD_F5 | 0 | 0.003 | 0.011 | 0.000 to 0.042 | No | No |
| HA_Som | HD_F6 | 0 | 0.000 | 0.003 | 0.000 to 0.000 | No | No |
| HD_F2 | HD_F6 | 0 | 0.006 | 0.016 | 0.000 to 0.061 | No | No |
| HD_F3 | HD_F6 | 0 | 0.004 | 0.011 | 0.000 to 0.039 | No | No |
| HD_F5 | HD_F6 | 0 | 0.000 | 0.003 | 0.000 to 0.000 | No | No |
| HD_F6 | HD_F7 | 0 | -0.000 | 0.003 | 0.000 to 0.000 | No | No |
| HA_Som | DB_F1 | 0 | 0.004 | 0.012 | 0.000 to 0.047 | No | No |
| HD_F4 | DB_F1 | 0 | 0.006 | 0.015 | 0.000 to 0.059 | No | No |
| HD_F5 | DB_F1 | 0 | 0.008 | 0.015 | 0.000 to 0.053 | No | No |
| HD_F7 | DB_F1 | 0 | 0.006 | 0.012 | 0.000 to 0.044 | No | No |
| HA_Som | DB_F2 | 0 | 0.002 | 0.008 | 0.000 to 0.027 | No | No |
| HD_F3 | DB_F2 | 0 | 0.001 | 0.005 | 0.000 to 0.016 | No | No |
| HD_F5 | DB_F2 | 0 | -0.000 | 0.002 | 0.000 to 0.000 | No | No |
| HD_F7 | DB_F2 | 0 | 0.000 | 0.003 | 0.000 to 0.000 | No | No |
| HA_Psy | DB_F3 | 0 | 0.000 | 0.003 | 0.000 to 0.000 | No | No |
| HA_Som | DB_F3 | 0 | 0.002 | 0.008 | 0.000 to 0.026 | No | No |
| HD_F2 | DB_F3 | 0 | -0.006 | 0.018 | -0.073 to 0.000 | No | No |
| HD_F4 | DB_F3 | 0 | 0.021 | 0.033 | 0.000 to 0.115 | No | No |
| HD_F5 | DB_F3 | 0 | 0.011 | 0.021 | 0.000 to 0.073 | No | No |
| HD_F6 | DB_F3 | 0 | -0.002 | 0.011 | -0.036 to 0.000 | No | No |
| HD_F7 | DB_F3 | 0 | 0.004 | 0.014 | 0.000 to 0.049 | No | No |
| HA_Psy | DB_F4 | 0 | -0.002 | 0.010 | -0.030 to 0.000 | No | No |
| HA_Som | DB_F4 | 0 | -0.002 | 0.010 | -0.033 to 0.000 | No | No |
| HD_F3 | DB_F4 | 0 | 0.017 | 0.026 | 0.000 to 0.090 | No | No |
| HD_F4 | DB_F4 | 0 | 0.009 | 0.022 | 0.000 to 0.081 | No | No |
| HD_F5 | DB_F4 | 0 | 0.000 | 0.003 | 0.000 to 0.000 | No | No |
| HD_F6 | DB_F4 | 0 | 0.021 | 0.031 | 0.000 to 0.097 | No | No |
| HD_F7 | DB_F4 | 0 | 0.001 | 0.006 | 0.000 to 0.011 | No | No |
| HA_Psy | SE | 0 | 0.000 | 0.004 | 0.000 to 0.000 | No | No |
| HA_Som | SE | 0 | 0.001 | 0.005 | 0.000 to 0.011 | No | No |
| HD_F2 | SE | 0 | -0.032 | 0.040 | -0.128 to 0.000 | No | No |
| HD_F3 | SE | 0 | 0.000 | 0.003 | 0.000 to 0.000 | No | No |
| HD_F4 | SE | 0 | -0.002 | 0.008 | -0.027 to 0.000 | No | No |
| HD_F5 | SE | 0 | -0.004 | 0.012 | -0.040 to 0.000 | No | No |
| HD_F7 | SE | 0 | -0.006 | 0.017 | -0.064 to 0.000 | No | No |
| DB_F1 | SE | 0 | -0.009 | 0.017 | -0.060 to 0.000 | No | No |
| DB_F2 | SE | 0 | -0.002 | 0.008 | -0.025 to 0.000 | No | No |
| DB_F3 | SE | 0 | 0.004 | 0.015 | 0.000 to 0.052 | No | No |
| DB_F4 | SE | 0 | -0.014 | 0.025 | -0.083 to 0.000 | No | No |
| HA_Som | AHI_log | 0 | -0.004 | 0.012 | -0.043 to 0.000 | No | No |
| HD_F2 | AHI_log | 0 | -0.003 | 0.011 | -0.039 to 0.000 | No | No |
| HD_F3 | AHI_log | 0 | -0.001 | 0.005 | -0.012 to 0.000 | No | No |
| HD_F4 | AHI_log | 0 | -0.001 | 0.006 | -0.020 to 0.000 | No | No |
| HD_F5 | AHI_log | 0 | -0.002 | 0.008 | -0.034 to 0.000 | No | No |
| HD_F6 | AHI_log | 0 | -0.000 | 0.004 | 0.000 to 0.000 | No | No |
| HD_F7 | AHI_log | 0 | -0.009 | 0.017 | -0.060 to 0.000 | No | No |
| DB_F1 | AHI_log | 0 | 0.002 | 0.007 | 0.000 to 0.022 | No | No |
| DB_F2 | AHI_log | 0 | -0.000 | 0.002 | 0.000 to 0.000 | No | No |
| DB_F3 | AHI_log | 0 | 0.000 | 0.003 | 0.000 to 0.000 | No | No |
| DB_F4 | AHI_log | 0 | -0.002 | 0.009 | -0.033 to 0.000 | No | No |
| SE | AHI_log | 0 | -0.004 | 0.012 | -0.045 to 0.000 | No | No |
| HA_Psy | ArI | 0 | -0.005 | 0.013 | -0.046 to 0.000 | No | No |
| HA_Som | ArI | 0 | -0.003 | 0.008 | -0.030 to 0.000 | No | No |
| HD_F2 | ArI | 0 | -0.005 | 0.014 | -0.049 to 0.000 | No | No |
| HD_F3 | ArI | 0 | 0.000 | 0.003 | 0.000 to 0.000 | No | No |
| HD_F4 | ArI | 0 | -0.000 | 0.003 | -0.000 to 0.000 | No | No |
| HD_F5 | ArI | 0 | 0.001 | 0.005 | 0.000 to 0.009 | No | No |
| HD_F6 | ArI | 0 | -0.002 | 0.008 | -0.025 to 0.000 | No | No |
| HD_F7 | ArI | 0 | 0.000 | 0.001 | 0.000 to 0.000 | No | No |
| DB_F1 | ArI | 0 | 0.000 | 0.003 | 0.000 to 0.000 | No | No |
| DB_F2 | ArI | 0 | 0.000 | 0.003 | 0.000 to 0.000 | No | No |
| DB_F3 | ArI | 0 | 0.003 | 0.010 | 0.000 to 0.038 | No | No |
| DB_F4 | ArI | 0 | 0.001 | 0.004 | 0.000 to 0.005 | No | No |
| HA_Psy | N3pct | 0 | -0.000 | 0.003 | 0.000 to 0.000 | No | No |
| HA_Som | N3pct | 0 | -0.001 | 0.004 | -0.005 to 0.000 | No | No |
| HD_F2 | N3pct | 0 | 0.007 | 0.018 | 0.000 to 0.066 | No | No |
| HD_F3 | N3pct | 0 | 0.000 | 0.004 | 0.000 to 0.000 | No | No |
| HD_F4 | N3pct | 0 | 0.001 | 0.007 | 0.000 to 0.023 | No | No |
| HD_F5 | N3pct | 0 | -0.001 | 0.006 | -0.017 to 0.000 | No | No |
| HD_F6 | N3pct | 0 | -0.000 | 0.007 | 0.000 to 0.000 | No | No |
| HD_F7 | N3pct | 0 | 0.002 | 0.008 | 0.000 to 0.024 | No | No |
| DB_F1 | N3pct | 0 | -0.000 | 0.003 | 0.000 to 0.000 | No | No |
| DB_F2 | N3pct | 0 | -0.016 | 0.025 | -0.084 to 0.000 | No | No |
| DB_F3 | N3pct | 0 | 0.001 | 0.008 | 0.000 to 0.023 | No | No |
| DB_F4 | N3pct | 0 | -0.017 | 0.029 | -0.092 to 0.000 | No | No |
| SE | N3pct | 0 | -0.004 | 0.016 | -0.062 to 0.000 | No | No |
| AHI_log | N3pct | 0 | 0.001 | 0.007 | 0.000 to 0.000 | No | No |
| HA_Psy | REMpct | 0 | -0.001 | 0.005 | -0.018 to 0.000 | No | No |
| HA_Som | REMpct | 0 | 0.000 | 0.005 | 0.000 to 0.000 | No | No |
| HD_F2 | REMpct | 0 | 0.001 | 0.009 | 0.000 to 0.004 | No | No |
| HD_F3 | REMpct | 0 | -0.004 | 0.013 | -0.043 to 0.000 | No | No |
| HD_F4 | REMpct | 0 | 0.000 | 0.005 | 0.000 to 0.000 | No | No |
| HD_F5 | REMpct | 0 | -0.001 | 0.004 | -0.009 to 0.000 | No | No |
| HD_F6 | REMpct | 0 | -0.013 | 0.025 | -0.086 to 0.000 | No | No |
| HD_F7 | REMpct | 0 | -0.016 | 0.025 | -0.083 to 0.000 | No | No |
| DB_F1 | REMpct | 0 | -0.011 | 0.022 | -0.078 to 0.000 | No | No |
| DB_F2 | REMpct | 0 | -0.013 | 0.022 | -0.074 to 0.000 | No | No |
| DB_F3 | REMpct | 0 | 0.008 | 0.021 | 0.000 to 0.075 | No | No |
| AHI_log | REMpct | 0 | -0.001 | 0.006 | 0.000 to 0.000 | No | No |
| N3pct | REMpct | 0 | 0.002 | 0.009 | 0.000 to 0.040 | No | No |
| HD_F4 | DB_F2 | <0.001 | 0.013 | 0.022 | 0.000 to 0.073 | Yes | No |

Based on 1,000 bootstrap resamples with participants sampled with replacement. Rows are ordered by the absolute edge weight. Edges set to zero by regularization are shown as 0. Intervals describe estimation precision and were not used to decide whether an edge is present. Of the 43 non-zero edges, 19 had intervals that excluded zero.

**Table S10. Comparison of all 14 covariance parameterizations for the primary latent profile analysis.**

| **Parameterization** | **Family** | **Classes** | **npar** | **logLik** | **BIC** | **Entropy** | **Smallest class, n (%)** | **Status** |
| --- | --- | --- | --- | --- | --- | --- | --- | --- |
| EII | Diagonal | 1 | 19 | -8,112.99 | 16,335.46 | 1.000 | 318 (100.0) | Eligible |
| EII | Diagonal | 2 | 38 | -7,793.37 | 15,805.69 | 0.808 | 126 (39.6) | Eligible |
| EII | Diagonal | 3 | 57 | -7,662.06 | 15,652.56 | 0.842 | 52 (16.4) | Eligible |
| EII | Diagonal | 4 | 76 | -7,567.81 | 15,573.53 | 0.857 | 30 (9.4) | Eligible |
| EII | Diagonal | 5 | 95 | -7,488.36 | 15,524.12 | 0.861 | 26 (8.2) | Eligible |
| EII | Diagonal | 6 | 114 | -7,460.00 | 15,576.88 | 0.832 | 14 (4.4) | Smallest class below 5% |
| VII | Diagonal | 1 | 19 | -8,112.99 | 16,335.46 | 1.000 | 318 (100.0) | Eligible |
| VII | Diagonal | 2 | 39 | -7,774.80 | 15,774.31 | 0.811 | 127 (39.9) | Eligible |
| VII | Diagonal | 3 | 59 | -7,617.16 | 15,574.28 | 0.847 | 46 (14.5) | Eligible |
| VII | Diagonal | 4 | 79 | -7,491.73 | 15,438.66 | 0.880 | 29 (9.1) | Eligible |
| VII | Diagonal | 5 | 99 | -7,430.63 | 15,431.71 | 0.854 | 23 (7.2) | Eligible |
| VII | Diagonal | 6 | 119 | -7,394.41 | 15,474.51 | 0.838 | 14 (4.4) | Smallest class below 5% |
| EEI | Diagonal | 1 | 36 | -8,112.99 | 16,433.41 | 1.000 | 318 (100.0) | Eligible |
| EEI | Diagonal | 2 | 55 | -7,739.70 | 15,796.31 | 0.857 | 117 (36.8) | Eligible |
| EEI | Diagonal | 3 | 74 | -7,585.37 | 15,597.14 | 0.875 | 56 (17.6) | Eligible |
| EEI | Diagonal | 4 | 93 | -7,497.62 | 15,531.11 | 0.891 | 24 (7.5) | Eligible |
| EEI | Diagonal | 5 | 112 | -7,399.92 | 15,445.19 | 0.888 | 22 (6.9) | Eligible |
| EEI | Diagonal | 6 | 131 | -7,364.67 | 15,484.17 | 0.864 | 14 (4.4) | Smallest class below 5% |
| VEI | Diagonal | 1 | 36 | -8,112.99 | 16,433.41 | 1.000 | 318 (100.0) | Eligible |
| VEI | Diagonal | 2 | 56 | -7,709.63 | 15,741.93 | 0.862 | 124 (39.0) | Eligible |
| VEI | Diagonal | 3 | 76 | -7,533.40 | 15,504.72 | 0.878 | 54 (17.0) | Eligible |
| VEI | Diagonal | 4 | 96 | -7,423.51 | 15,400.19 | 0.898 | 28 (8.8) | Eligible |
| VEI | Diagonal | 5 | 116 | -7,328.63 | 15,325.66 | 0.897 | 20 (6.3) | Eligible |
| VEI | Diagonal | 6 | 136 | -7,279.17 | 15,341.97 | 0.894 | 13 (4.1) | Smallest class below 5% |
| EVI | Diagonal | 1 | 36 | -8,112.99 | 16,433.41 | 1.000 | 318 (100.0) | Eligible |
| EVI | Diagonal | 2 | — | — | — | — | — | Not estimable |
| EVI | Diagonal | 3 | — | — | — | — | — | Not estimable |
| EVI | Diagonal | 4 | — | — | — | — | — | Not estimable |
| EVI | Diagonal | 5 | — | — | — | — | — | Not estimable |
| EVI | Diagonal | 6 | — | — | — | — | — | Not estimable |
| VVI | Diagonal | 1 | 36 | -8,112.99 | 16,433.41 | 1.000 | 318 (100.0) | Eligible |
| VVI | Diagonal | 2 | — | — | — | — | — | Not estimable |
| VVI | Diagonal | 3 | — | — | — | — | — | Not estimable |
| VVI | Diagonal | 4 | — | — | — | — | — | Not estimable |
| VVI | Diagonal | 5 | — | — | — | — | — | Not estimable |
| VVI | Diagonal | 6 | — | — | — | — | — | Not estimable |
| EEE | Ellipsoidal | 1 | 189 | -7,143.72 | 15,376.47 | 1.000 | 318 (100.0) | Eligible |
| EEE | Ellipsoidal | 2 | 208 | -7,021.27 | 15,241.05 | 1.000 | 128 (40.3) | Eligible |
| EEE | Ellipsoidal | 3 | 227 | -6,991.54 | 15,291.07 | 0.960 | 28 (8.8) | Eligible |
| EEE | Ellipsoidal | 4 | 246 | -6,957.24 | 15,331.94 | 0.968 | 10 (3.1) | Smallest class below 5% |
| EEE | Ellipsoidal | 5 | — | — | — | — | — | Not estimable |
| EEE | Ellipsoidal | 6 | — | — | — | — | — | Not estimable |
| VEE | Ellipsoidal | 1 | 189 | -7,143.72 | 15,376.47 | 1.000 | 318 (100.0) | Eligible |
| VEE | Ellipsoidal | 2 | 209 | -7,050.98 | 15,306.22 | 0.744 | 155 (48.7) | Entropy below 0.80 |
| VEE | Ellipsoidal | 3 | 229 | -7,013.18 | 15,345.86 | 0.840 | 37 (11.6) | Eligible |
| VEE | Ellipsoidal | 4 | 249 | -6,955.69 | 15,346.13 | 0.880 | 11 (3.5) | Smallest class below 5% |
| VEE | Ellipsoidal | 5 | 269 | -6,924.94 | 15,399.88 | 0.903 | 12 (3.8) | Smallest class below 5% |
| VEE | Ellipsoidal | 6 | — | — | — | — | — | Not estimable |
| EVE | Ellipsoidal | 1 | 189 | -7,143.72 | 15,376.47 | 1.000 | 318 (100.0) | Eligible |
| EVE | Ellipsoidal | 2 | — | — | — | — | — | Not estimable |
| EVE | Ellipsoidal | 3 | — | — | — | — | — | Not estimable |
| EVE | Ellipsoidal | 4 | — | — | — | — | — | Not estimable |
| EVE | Ellipsoidal | 5 | — | — | — | — | — | Not estimable |
| EVE | Ellipsoidal | 6 | — | — | — | — | — | Not estimable |
| VVE | Ellipsoidal | 1 | 189 | -7,143.72 | 15,376.47 | 1.000 | 318 (100.0) | Eligible |
| VVE | Ellipsoidal | 2 | — | — | — | — | — | Not estimable |
| VVE | Ellipsoidal | 3 | — | — | — | — | — | Not estimable |
| VVE | Ellipsoidal | 4 | — | — | — | — | — | Not estimable |
| VVE | Ellipsoidal | 5 | — | — | — | — | — | Not estimable |
| VVE | Ellipsoidal | 6 | — | — | — | — | — | Not estimable |
| EEV | Ellipsoidal | 1 | 189 | -7,143.72 | 15,376.47 | 1.000 | 318 (100.0) | Eligible |
| EEV | Ellipsoidal | 2 | 361 | -6,779.72 | 15,639.55 | 0.954 | 99 (31.1) | Eligible |
| EEV | Ellipsoidal | 3 | 533 | -6,451.65 | 15,974.47 | 0.977 | 52 (16.4) | Eligible |
| EEV | Ellipsoidal | 4 | 705 | -6,352.66 | 16,767.56 | 0.978 | 43 (13.5) | Eligible |
| EEV | Ellipsoidal | 5 | 877 | -6,277.21 | 17,607.74 | 0.976 | 38 (11.9) | Eligible |
| EEV | Ellipsoidal | 6 | 1049 | -6,105.67 | 18,255.74 | 0.987 | 21 (6.6) | Eligible |
| VEV | Ellipsoidal | 1 | 189 | -7,143.72 | 15,376.47 | 1.000 | 318 (100.0) | Eligible |
| VEV | Ellipsoidal | 2 | 362 | -6,718.03 | 15,521.93 | 0.951 | 109 (34.3) | Eligible |
| VEV | Ellipsoidal | 3 | 535 | -6,480.78 | 16,044.26 | 0.965 | 67 (21.1) | Eligible |
| VEV | Ellipsoidal | 4 | 708 | -6,327.63 | 16,734.78 | 0.979 | 40 (12.6) | Eligible |
| VEV | Ellipsoidal | 5 | 881 | -6,229.42 | 17,535.21 | 0.980 | 37 (11.6) | Eligible |
| VEV | Ellipsoidal | 6 | 1054 | -6,140.12 | 18,353.44 | 0.985 | 12 (3.8) | Smallest class below 5% |
| EVV | Ellipsoidal | 1 | 189 | -7,143.72 | 15,376.47 | 1.000 | 318 (100.0) | Eligible |
| EVV | Ellipsoidal | 2 | — | — | — | — | — | Not estimable |
| EVV | Ellipsoidal | 3 | — | — | — | — | — | Not estimable |
| EVV | Ellipsoidal | 4 | — | — | — | — | — | Not estimable |
| EVV | Ellipsoidal | 5 | — | — | — | — | — | Not estimable |
| EVV | Ellipsoidal | 6 | — | — | — | — | — | Not estimable |
| VVV | Ellipsoidal | 1 | 189 | -7,143.72 | 15,376.47 | 1.000 | 318 (100.0) | Eligible |
| VVV | Ellipsoidal | 2 | — | — | — | — | — | Not estimable |
| VVV | Ellipsoidal | 3 | — | — | — | — | — | Not estimable |
| VVV | Ellipsoidal | 4 | — | — | — | — | — | Not estimable |
| VVV | Ellipsoidal | 5 | — | — | — | — | — | Not estimable |
| VVV | Ellipsoidal | 6 | — | — | — | — | — | Not estimable |

All 14 covariance parameterizations available in mclust were fitted for one to six profiles, giving 84 models. Selection was restricted to the six parameterizations with diagonal covariance matrices, consistent with the local independence assumption of latent profile analysis, and required entropy of at least 0.80 and a smallest class of at least 5% of the sample, applied together. For the one-class model all parameterizations within a family return an equivalent solution. Models marked as not estimable returned no fit, reflecting the ratio of parameters to sample size. Ellipsoidal parameterizations are reported for completeness but were not eligible for selection. npar, number of estimated parameters.

**Supplementary Figures**

**Figure S1.** Observed distributions of the 17 final network variables (N = 318).

Histograms use the Freedman-Diaconis rule for bin width. Values are shown on the original measurement scale, except for the AHI node, which is log(AHI + 1). Variable codes are defined in Table S1A.

**Figure S2.** Nonparametric bootstrap 95% intervals for all 136 possible edge weights.

Based on 1,000 resamples with participants sampled with replacement; edges are ordered by weight. Dark blue marks the 19 non-zero edges whose intervals exclude zero, light blue the 24 non-zero edges whose intervals include zero, and grey the 93 edges set to zero by regularization. Intervals describe estimation precision and are not multiplicity-adjusted significance tests.

**Figure S3.** Eight-indicator sensitivity latent profile solution.

(A) Standardized means for the eight aggregate indicators. (B) The same classes shown across the original 18 indicators, with the dashed line separating the two modalities. Classes are numbered independently of the main solution and do not correspond to C1 through C5; agreement between the two classifications is reported in Tables S6B and S6C.

**Figure S4.** Main and sensitivity networks under a unified layout and edge scale.

All four networks use the node placement and edge scaling of the main network so that structures can be compared directly. Blue edges are positive and red edges are negative regularized partial correlations. Variable codes are defined in Table S1A.
